# *Haematoxylon campechianum* Extract Ameliorates Neuropathic Pain via Inhibition of NF-κB/TNF-α/NOX/iNOS Signalling Pathway in a Rat Model of Chronic Constriction Injury

**DOI:** 10.3390/biom10030386

**Published:** 2020-03-02

**Authors:** Mansour Sobeh, Mona F. Mahmoud, Samar Rezq, Mohamed A. O. Abdelfattah, Islam Mostafa, Amira E. Alsemeh, Assem M. El-Shazly, Aziz Yasri, Michael Wink

**Affiliations:** 1 Institute of Pharmacy and Molecular Biotechnology, Heidelberg University, 69120 Heidelberg, Germany; 2 AgroBioSciences Research Division, Mohammed VI Polytechnic University, Lot 660–Hay MoulayRachid, Ben-Guerir 43150, Morocco; aziz.yasri@um6p.ma; 3 Department of Pharmacology and Toxicology, Faculty of Pharmacy, Zagazig University, Zagazig 44519, Egypt; mona_pharmacology@yahoo.com (M.F.M.); samar_rezq@yahoo.com (S.R.); 4 College of Engineering and Technology, American University of the Middle East, Egaila 54200, Kuwait; mohamed.abdelmoety@aum.edu.kw; 5 Department of Pharmacognosy, Faculty of Pharmacy, Zagazig University, Zagazig 44519, Egypt; islam_mostafa_elbaz@yahoo.com (I.M.); assemels2002@yahoo.co.uk (A.M.E.-S.); 6 Department of Anatomy and Embryology, Faculty of Medicine, Zagazig University, Zagazig 44519, Egypt; dr_amira_2008@yahoo.com

**Keywords:** *Haematoxylon campechianum*, inflammation, neuropathic pain, NF-κB/TNF-α/NOX/iNOS, polyphenols

## Abstract

In this study, the phytochemical composition and the possible prophylactic effects of an aqueous ethanol extract of *Haematoxylon campechianum* flowers (HCF) on peripheral neuropathic pain in a chronic constriction injury (CCI) rat model are investigated. Rats with induced CCI were subjected to neuropathic pain behaviour tests and evaluated by chemical, thermal, and mechanical sensation tests and functional recovery of the brain stem and sciatic nerve at 7- and 14-day intervals. The effect of the extract on acute pain and inflammation is also investigated. The extract exerted both peripheral and central analgesic and anti-inflammatory properties in addition to antipyretic effects that are clear from targeting COX, LOX and PGE. It was found that CCI produced significant thermal and mechanical hyperalgesia, cold allodynia and deleterious structural changes in both sciatic nerve and brain stem. Treatments with HCF extract significantly improved cold and thermal withdrawal latency, mechanical sensibility and ameliorated deleterious changes of sciatic nerve and brain stem at different dose levels. The extract also ameliorated oxidative stress and inflammatory markers in brain stem and sciatic nerve. It suppressed the apoptotic marker, p53, and restored myelin sheath integrity. The effects of HCF extract were more potent than pregabalin. Fifteen secondary metabolites, mainly gallotannins and flavonoids, were characterized in the extract based on their retention times and MS/MS data. The identified phenolic constituents from the extract could be promising candidates to treat neuropathic pain due to their diverse biological activities, including antioxidant, anti-inflammatory and neuroprotective properties.

## 1. Introduction

*Haematoxylon campechianum* (logwood) is a common tree belonging to the family Fabaceae. It is native to Mexico and South America and commonly used as an anti-spasmodic and astringent to treat diarrhoea and dysentery [1,2]. Several authors have studied its secondary metabolites. From the heartwood of the plant, Escobar-Ramos et al. identified two chalcones, namely sappanchalcone and 3-deoxysappanchalcone, and the homoisoflavonoids, hematoxylol A, 4-*O*-methylhematoxylol and hematoxin [2]. Other compounds were isolated from the bark extract, such as the 5′-*O*-methyl-7′-ethyl ester of *p*-dehydrodigallic acid, genistein, 3-methoxyquercetin and ethyl gallate. The latter exhibited anti-microbial activity against *Xanthomonas campestris* and *Mycobacterium tuberculosis* [3,4]. Imperatorin, marmesin, umbelliferone, 2,6-bis-*O*-digalloyl-3-*O*-galloylglucose and 2-*O*-trigalloyl-1,3,4,6-tetrakis-*O*-galloylglucose, among others, were detected in the leaves of the *campechianum* species [5].

Hematoxylin, along with some lignans, flavonoids and homoisoflavonoids such as epihematoxylol B, 10-*O*-methylhematoxylol B and 10-*O*-methylepihematoxylol B, were identified in the heartwood and reported to inhibit protein tyrosine kinase [1], a key mediator in the acute inflammatory responses. Moreover, the twigs produced several phenolic acids, flavonoids and galloylglucosides [6]. Hematoxylin is an interesting protein tyrosine kinase inhibitor with potential use as a chemotherapeutic agent for cancer treatment [7] and as an anti-inflammatory agent [8]. Moreover, it reduced melanin production with subsequent skin whitening [9].

In this work, the active constituents of an aqueous ethanol extract from *H. campechianum* flowers were characterized using HPLC-MS/MS. The possible antipyretic, anti-nociceptive as well as, the central and peripheral anti-inflammatory activities of the extract were explored in different animal models. The potential of the extract to alleviate neuropathic pain in a rat model of chronic constriction (CCI) injury was also investigated. To gain insights into the molecular mechanism of action of the extract, the levels of different inflammatory and oxidative stress markers, including prostaglandin E2 (PGE2), inducible nitric oxide synthase (iNOS), cyclooxygenase-2 (COX2), 5-lipoxygenase (LOX), p53, TNF-α, NF-κB, catalase and NADPH oxidase 1 (NOX1), were investigated. Molecular docking was performed to confirm the inhibitory potential of some of the extracts’ components to suppress p53 protein. Moreover, the inhibition of COX1, COX2, and LOX by the extract, as well as its antioxidant activity, were determined using in vitro assays.

## 2. Materials and Methods

### 2.1. Plant Material and Extraction

Flowers of *Haematoxylon campechianum* were collected in March 2018 from the vicinity of Benha (Tahla–Mit El-Attar road), Qalubiya province, Egypt (Location: 30.422352N, 31.139488E) and a voucher specimen (HCF-103) is kept in the herbarium of the Pharmacognosy Department at Faculty of Pharmacy, Zagazig University, Egypt. The flowers (400 g) were dried in the shade and ground to fine powder by an electric mill. The powder was extracted twice at room temperature by aqueous ethanol (70%). The obtained extracts were combined, filtered and concentrated at 40 °C to yield 76 g of the flowers total extract (yield; 19% *w/w*). The concentrated extract was then defatted with hexane, frozen, lyophilized and stored in a refrigerator for chemical and biological studies.

### 2.2. LC-MS/MS Analysis

The process of LC-MS/MS profiling of *H. campechianum* flower extract was performed as previously described by Sobeh et al. [10].

### 2.3. Animals

Swiss albino mice (20–25 g) and adult male Wistar rats (140–160 g) were obtained from the Faculty of Veterinary Medicine, Zagazig University, Egypt. Different groups of animals were used in each experiment. The animals were housed in cages with wood shave bedding and kept at 22 °C with 50% ± 10% humidity and a 12-h light/dark cycle. The animals were supplied with water and regular chow diet ad libitum. Animal handling procedures and experimental design were approved by the Ethical Committee for Animal Handling at Zagazig University (ECAH ZU), Faculty of Pharmacy, Zagazig University, Egypt that comes in accordance with the recommendations of the Weather all report (Approval number: ZU-IACUC/3/F/115/2018).

### 2.4. Antioxidant, Antipyretic, Anti-Nociceptive and Anti-Inflammatory Experiments

In vitro antioxidant activities, inhibition of cyclooxygenases (COXs) and lipoxygenase (LOX) and in vivo antipyretic, anti-nociceptive, and anti-inflammatory activities were carried out as described by Sobeh et al. [11]. The inhibitory concentration 50 (IC_50_) value (μM), which is the concentration causing 50% COX enzymes inhibition in μM, was detected using an enzyme immunoassay (EIA) kit (Cayman Chemical, AnnArbor, MI, USA), according to the manufacturer’s instruction, for the extract and compared to that of celecoxib, indomethacin and diclofenac sodium. Additionally, the IC_50_ (COX-1)/IC_50_ (COX-2) ratio that represents the COX2 selectivity index (SI values) was also computed. The concentration of the extract in μM (IC_50_) that achieved 50% inhibition of lipoxygenase enzyme (LOX) using a lipoxygenase inhibitor screening assay kit (Cayman Chemical, AnnArbor, MI, USA) was calculated using the LOX inhibitor, zileuton, as a reference standard.

### 2.5. Induction of Neuropathic Pain by Chronic Constriction Injury

The chronic constriction injury in the rats’ sciatic nerve was unilaterally induced, as previously reported by Bennett and Xie [12]. Following thiopental anesthesia (50 mg/kg, i.p.), the sciatic nerve of the right limb was exposed under aseptic conditions. Four loose, 1-mm apart ligatures (4/0 silk suture) were placed along the nerve to induce CCI. The wound was stitched close, and the animals were allowed to recover in their cages. In the sham group, the sciatic nerve of the right limb was just exposed without constriction. Behavioural tests were carried out one day before the surgical procedure and at days 7 and 14 following the surgery. The animal groups received the different treatments on a daily basis after the surgery and for the whole experiment duration. Animals were sacrificed after performing the last behavioural test on day 14; then, blood and tissue samples were collected for measuring different biochemical markers.

### 2.6. Behavioural Tests

Behavioural tests were blinded in a way that the experimenter who conducted the tests was not aware of the nature of the experimental manipulation.

### 2.7. Mechanical Hyperalgesia (Pin Prick Test)

Mechanical hyperalgesia was evaluated by the pin prick test, as described in Erichsen and Blackburn-Munro [13]. Briefly, a bent gauge needle was used to touch the injured hind paw without skin penetration. The duration of the resulted paw withdrawal response was recorded in seconds, giving 0.5 s for the brief normal response. The cut-off time was set to be 20 s.

### 2.8. Mechanical Dynamic Allodynia (Paint Brush Test)

Allodynia refers to the painful response to a stimulus that is normally painless. This model evaluates the response of the injured paw to a mechanical stimulus induced by a paint brush [14,15]. The animal was placed on an elevated wire mesh floor covered with a glass cage. The plantar area of the injured hind paw was rubbed from heel to toes using a paint brush for five times separated by a 5 s interval. The number of withdrawals (between 0 and 5) was recorded. The procedure was repeated twice with a 5 min gap in between (total 15 trials). The scores of all trials were summed to obtain a dynamic allodynia score with a minimum of 0 and a maximum of 15.

### 2.9. Heat Hyperalgesia (Hot Plate Test)

The hot plate test was used to assess the hyperalgesia response to heat, as reported by Jain et al. [16]. The animal was placed on a hot plate set with a temperature of 52.5 ± 1.0 °C. The time taken for the animal to show the first sign of hyperalgesia (paw licking, paw withdrawal, shaking or jumping) was recorded in seconds using a cut-off value of 20 sec.

### 2.10. Paw Cold Allodynia (Acetone Drop Test)

The response towards cold allodynia was evaluated by the acetone drop test in which the injured paw was exposed to a cold stimulus obtained by spraying a drop (100 μL) of acetone. Animals were monitored for 20 s and the response was graded by a four-point scale based on the following: 0 = no response; 1 = quick withdrawal, stamp or flick of the paw; 2 = prolonged withdrawal or repeated flicking; and 3 = repeated paw flicking with paw licking. The test was repeated three times, with 5 min intervals in between. The three trial scores were summed to give the final score (from 0 to 9) [17].

### 2.11. Histopathological Examination

Histopathological examination was carried out according to Bancroft and Gamble [18]. Brains and sciatic nerves were collected (n = 5 each) and placed in 10% neutral formalin then embedded in paraffin. Sections of 5 μm thickness were fixed on glass slides, deparaffinised by xylene, and stained with hematoxylin and eosin stain (H&E). The sections were examined by light microscopy (LEICA ICC50 W, Leica Microsystems (Schweiz) AG, 9435 Heerbrugg, Switzerland), and the damage of myelin sheath was estimated (6 sections/animal) according to the Nerve Injury Scoring System (NISS), where 1 = normal or mild degeneration, 2 = moderate degeneration (<50% damage in nerve tissue) and 3 = diffuse degeneration or demyelination (> 50% damage in nerve tissue) [19].

### 2.12. Immunohistochemical Staining of p53

The p53 expression was detected using the streptavidin–biotin complex immunoperoxidase system. Specimen sections were dewaxed and incubated in hydrogen peroxide (0.1%) for 30 min to block the endogenous peroxidases then rinsed thrice with phosphate-buffered saline (PBS). The antigen was retrieved by microwave treatment (20 min, 0.01 M/L citrate buffer, pH 6). Following demasking, the slides were kept overnight with rabbit monoclonal p53 antibody (monoclonal mouse anti-human p53 protein; clone DO-7; N1581; 10041283; 11 mL), diluted as 1:200 and applied to the sections for 12 h at 4 °C. The sections were washed with PBS and incubated with biotinylated anti-rabbit antibody (versal kits, Zymed Laboratories) at room temperature for 30 min. The sections were then counterstained with Mayer’s hematoxylin, dehydrated, and mounted. In the negative control, the primary antibody was replaced with PBS. The presence of neurons with brown-stained nuclei was considered as a positive reaction for p53 [20]. The Image J analysis software (Fiji image j; 1.51 n, NIH, USA) was used to count the number of p53 positive neurons in 3 non-overlapping perceptive fields at 200x magnification from 6 sections/animal, (5 animals/group). Data were expressed as mean percentage values of the p53 immunopositive neurons with dark brown nuclei numbers to that of the total neurons in the field.

### 2.13. Immunohistochemical Staining of Glial Fibrillar Acidic Protein (GFAP)

Immunohistochemical localization of GFAP in the brain stem was performed to inspect the allocation of astrocytes and their response to neuronal degeneration or injury as it is the most commonly used method to examine the distribution of astrocytes [21]. The modified avidin–biotin immunoperoxidase technique for GFAP was applied to demonstrate the astrocytes. Primary anti-GFAP antibody goat polyclonal IgG, anti-rat produced by DakoCytomation was used. Working dilution was 1:1000. GFAP-containing cells (astrocytes) appeared brown [22]. The positive results were indicated by a brown coloration of the cell membrane and cytoplasm of the astrocytes. The Image J analysis software (Fiji Image J; 1.51 n, NIH, USA) was used to estimate the area percentage of GFAP reactivity in 3 non-overlapping perceptive fields at 400× magnification from 6 sections/animal, (5 animals/group). Then the percent area relative to the field was calculated.

### 2.14. Staining of Sciatic Nerves with Osmic Acid

For the morphometrical assessment of the sciatic nerve, osmic acid was used to stain the myelin sheath of the nerve, as previously described [21]. Briefly, the specimens were fixed in 4% paraformaldehyde, and then placed in 2% osmic acid solution for 3 days. Then after, the specimens were transferred to 75% alcohol for 2 h then embedded in paraffin. The tissues were cut into 3-µm sections with a conventional microtome. Myelin sheaths were stained with a dark brown colour upon examination by light microscopy (LEICA ICC50 W, Leica Microsystems (Schweiz) AG, 9435 Heerbrugg, Switzerland). The integrity of the sciatic nerve obtained from the different groups was evaluated by determining the ratio of the myelinated area to the total nerve fibre area (including the myelin sheath). Additionally, the percentage of degenerated nerve fibres (fibres that lack the normal concentric lamellar structure of the myelin sheath or has myelin invagination into the axon and axonal swelling) to the total count of nerves was also estimated by a histologist who did not know the treatments.

### 2.15. Chronic Ulcerogenic Activity

By the end of the treatment period, animals were euthanized and their stomachs were collected to investigate the ulcerative potential of the extract on the stomach. The obtained stomachs were washed with normal saline solution (0.9%) and examined for ulceration. The ulcer severity scores were recorded, as previously described by Kulkarni [22] and Sakr et al. [23], where zero was given for normally-coloured stomach, 0.5 for red coloration, 1 for a spot ulcer, 1.5 for haemorrhagic streaks, 2 for an ulcer with a diameter of 3 to 5 mm, and 3 for an ulcer with a diameter larger than 5 mm. The ulcer index (UI) was determined using the equation: [UI = UN + US + UP × 10^−1^], where UN is the average number of ulcers, US is the severity scores average, and UP is the percentage of animals showed an ulcer. Celecoxib and indomethacin were used as reference standards.

### 2.16. Biochemical Measurements

The progression of chronic neuropathic pain involves both peripheral and central components; thus the anti-inflammatory potential of the extract was evaluated in the sciatic nerve and in the brain stem as well [24]. Tissues of the brain stem and sciatic nerve obtained from the different animals were homogenized in PBS and centrifuged at 14,000 rpm for 20 min at 4 °C. Bradford assay (Bio-Rad, 1000 Alfred Nobel Drive, Hercules, California 94547, USA) was used to estimate the protein content of the supernatant. ELISA kits obtained from Cusabio (TX, USA) were used to detect NADPH oxidase 1 (NOX1), cyclooxygenase-2 (COX-2), 5-lipoxygenase 1 (5-LOX), catalase, TNF-α, and NF-κB according to the manufacturer’s instructions. Inducible nitric oxide synthase (iNOS) and Prostaglandin E2 (PGE2) were determined using rat ELISA kits obtained from MyBiosource (San Diego, CA, USA) and Cayman (Michigan, USA), respectively, according to the manufacturers’ instructions.

### 2.17. Molecular Docking

The potential of some of the extract’s individual components to interfere with the p53 protein was evaluated by docking them to the crystallized DNA binding domain of p53 (PDB ID: 4QO1). Chemical structures of the compounds were drawn using the builder tool of the molecular operating environment (MOE) software (2013.08; Chemical Computing Group Inc., Montreal, QC, Canada, H3A 2R7, 2016). Energy minimization of the compounds was done using the molecular mechanic force-field, mmff94x, and their ionization state at physiological conditions was then assigned. The crystallised structure of the p53 DNA binding domain was downloaded from the protein data bank website (www.pdb.org) and was assigned the geometry and protonation state. Docking experiments were performed using the placement method triangle matcher and the scoring function London dG to estimate the binding free energy of the docked compounds.

### 2.18. Statistical Analysis

Data analysis was done by GraphPad Prism version 5 (GraphPad Software, San Diego, CA) and was expressed as the mean ± S.E.M. Results were analyzed using one-way analysis of variance (ANOVA) or repeated-measures analysis of variance (RM-ANOVA). The difference among groups was detected using Student’s *t*-test. Tukey’s multiple comparison test was used when appropriate for *post hoc* analysis. Statistically significant results were considered at *p*-values lower than 0.05.

## 3. Results

### 3.1. Chemical Composition of H. campechianum Flowers

**Figure 1:**
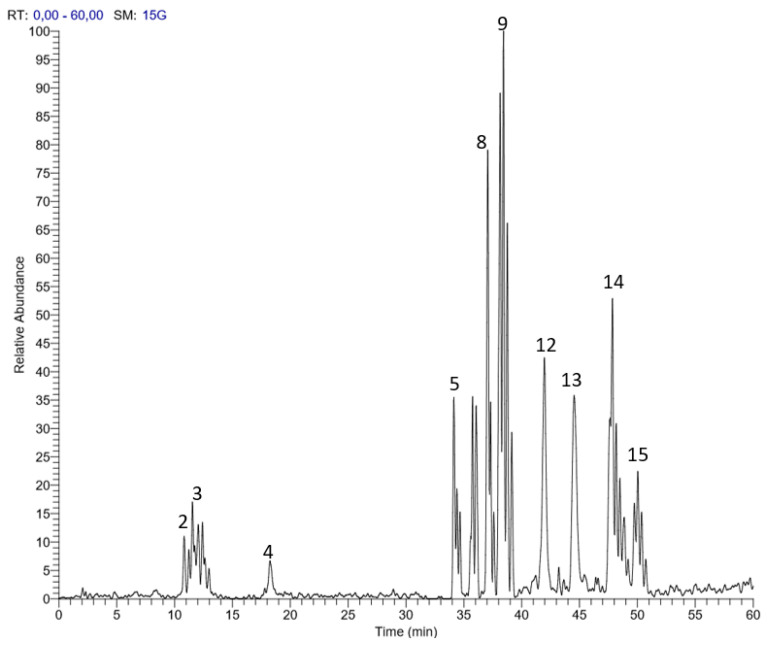
LC–MS profile of *Haematoxylon campechianum* flower extract.

Altogether, 15 secondary metabolites were characterized in the flower extract based on retention times and the MS/MS data (Figure 1 and Table 1). The tentative identification revealed two structurally-related hydrolysable tannins (gallotannins) and flavonoids. Compound 14 (retention time 47.70 min) exhibited a parent ion [M-H]^−^ at m/z 599 and was tentatively identified as isorhamentin galloyl-pentoside based on the loss of 152 amu (galloyl moiety fragmentation) and 132 amu (pentose sugar fragmentation) moieties.

### 3.2. Inhibition of Cyclooxygenases, Lipoxygenase, and Total Antioxidant Capacity (TAC)

As shown in Table 2, the HCF extract inhibited both COX1 and COX2 in vitro with more selectivity towards COX2 (SI = 16.9). Interestingly, the extract showed selectivity for COX2 compared to diclofenac (SI = 5.57). As for LOX, the extract showed similar potency to that of zileuton, the reference LOX enzyme inhibitor. Further, the total antioxidant capacity (TAC) of the extract showed to be higher than that of ascorbic acid (Table 2).

### 3.3. Effect of the Extract on Carrageenan-Induced Edema in Rats

**Figure 2:**
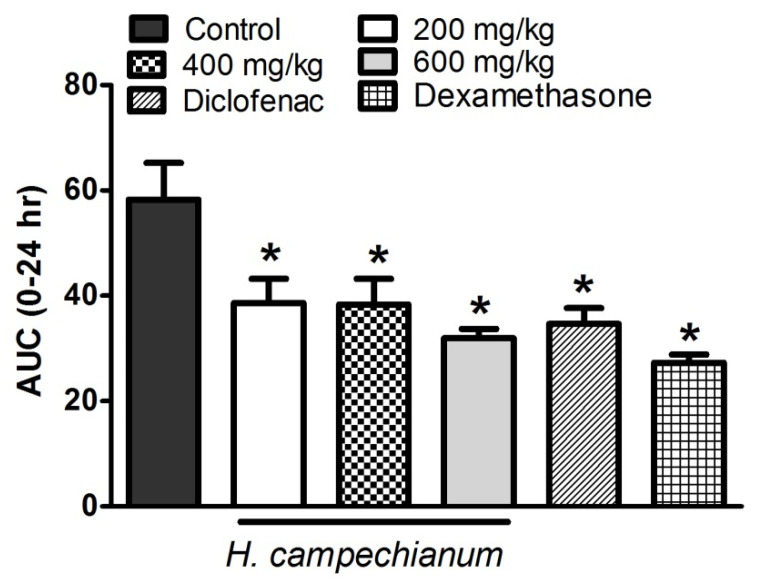
Effect of *H. campechianum* flower extract (p.o., 200, 400 and 600 mg/kg), diclofenac (p.o., 20 mg/kg) and dexamethasone (p.o., 2 mg/kg) on carrageenan (1%)-induced edema in paws of rats. Edema thickness was measured in mm before, hourly for 5 h, and finally at 24 h after carrageenan injection. Data shows the AUC0-24 and is expressed as mean values ± S.E.M (n = 5). * *p* < 0.05 vs. control values.

Sub-planter injection of 1% carrageenan (0.1 mL) resulted in an inflammatory response demonstrated as an increased paw thickness, which was measured at hourly intervals for 5 h and finally at 24 h after the injection. The strongest response was observed 2 h after the injection, shown as 3.89 ± 0.27 mm paw thickness over the baseline readings (paw thickness measured prior to carrageenan injection). Treatment with the extract (p.o., 200 and 400 mg/kg) 1 h before carrageenan injection attenuated the edema development as demonstrated by a reduction in the AUC0-24 values by nearly 34% of the control values. An extract dose of 600 mg/kg resulted in a slightly higher reduction of edema thickness (45%) compared to the control. This effect was comparable to that of diclofenac (p.o., 20 mg/kg). The effect was also comparable to that of the steroidal anti-inflammatory drug dexamethasone (p.o., 2 mg/kg), which inhibited the edema formation by 53% (Figure 2).

### 3.4. Effect of the Extract on Leukocyte Migration to the Peritoneal Cavity in Mice

**Figure 3:**
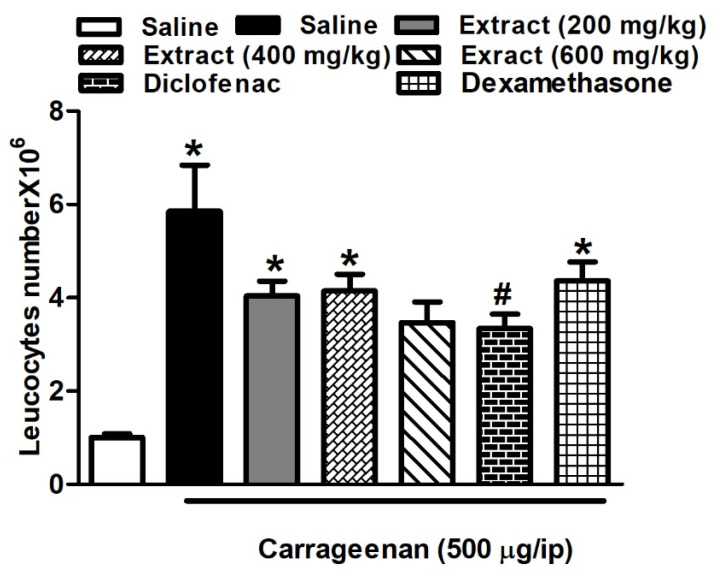
Carrageenan effect on leukocyte migration to the peritoneal cavity of mice (total number × 10^6^), with and without 1 h earlier treatment with *H. campechianum* flower extract in three different doses (p.o., 200, 400 and 600 mg/kg), diclofenac (p.o., 20 mg/kg) and dexamethasone (p.o., 2 mg/kg). Data are presented as mean values ± S.E.M (n = 5–7). * *p* < 0.05 vs. saline values, # *p* < 0.05 vs. control (carrageenan-treated group).

Intraperitoneal injection of 0.1 mL carrageenan (500 μg/cavity) stimulated the migration of leukocytes to the peritoneal cavity in mice, observed as an increase in the total number of leukocytes relative to that of the saline-treated mice (5.85 ± 0.99 vs. 1.01 ± 0.74 leukocytes × 10^6^ mL^−1^). Mice treated with the three doses of the extract 1 h before the carrageenan challenge decreased total leukocytes numbers. Noteworthy, the effect of the lower doses was comparable to that of dexamethasone (p.o., 2 mg/kg). The highest dose (p.o., 600 mg/kg) and diclofenac (p.o., 20 mg/kg) showed similar responses (Figure 3).

### 3.5. Effect of the Extract on Vascular Permeability in Mice

**Figure 4:**
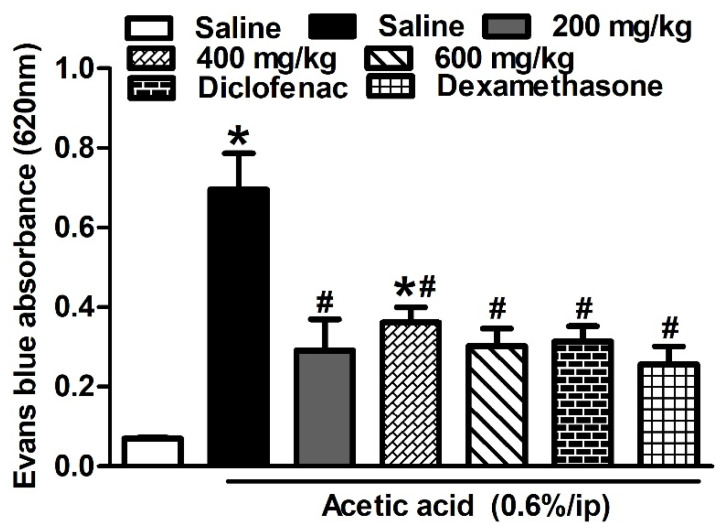
Effect of *H. campechianum* flower extract tested doses (p.o., 200, 400 and 600 mg/kg) on vascular permeability induced by acetic acid in mice relative to diclofenac (p.o., 20 mg/kg) and dexamethasone (p.o., 2 mg/kg). Evans blue dye absorbed in the peritoneal cavity was measured to indicate the degree of inflammation. The values are presented as mean values ± SEM (n = 5–6). * *p* < 0.05 compared to the saline group. # *p* < 0.01 compared to the control group (treated with acetic acid only).

Injecting mice with acetic acid (i.p., 0.6%) resulted in an increased vascular permeability evidenced by the greater Evans blue absorbance values of the peritoneal cavity exudates compared to those obtained from the mice injected with saline (0.70 ± 0.09 vs. 0.07± 0.003; *p* < 0.001). Mice treated with the HCF extract (p.o., 200, 400 and 600 mg/kg) 1 h before acetic acid injection showed a 48–59% reduction in vascular permeability, an effect that is comparable to that of diclofenac (p.o., 20 mg/kg) and dexamethasone (p.o., 2 mg/kg). The different doses of the extract had similar effects on vascular permeability (Figure 4).

### 3.6. Effect of the Extract on Writhing Response in Mice

**Figure 5:**
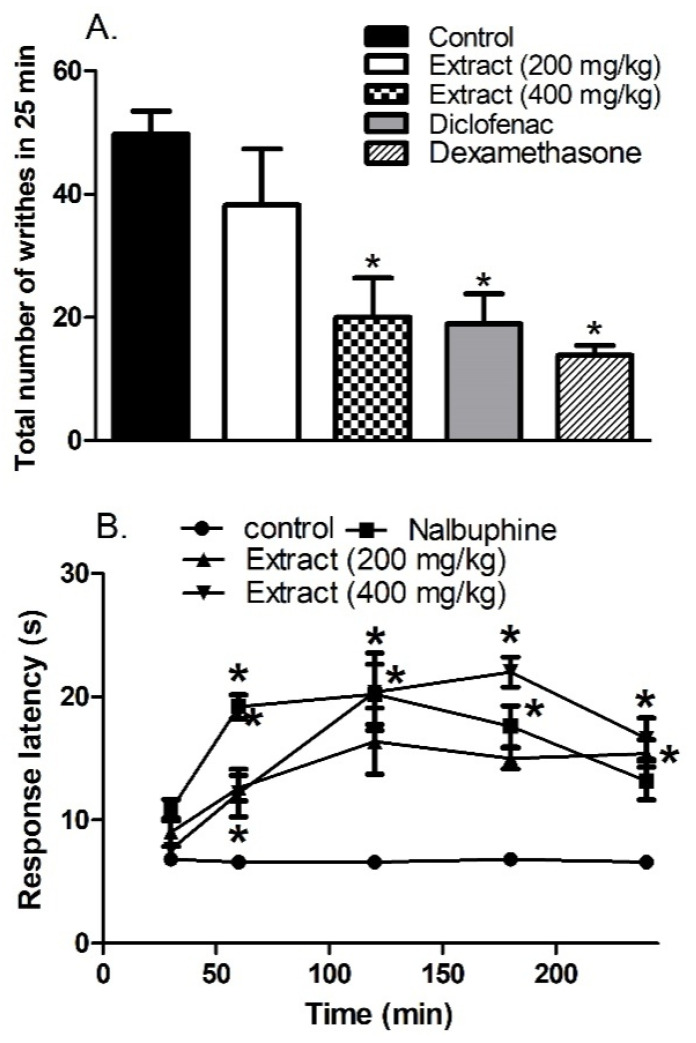
(**A**). Effects of *H. campechianum* flower extract (p.o., 200 and 400 mg/kg), diclofenac (p.o., 20 mg/kg) and dexamethasone (p.o., 2 mg/kg) on writhing induced by 0.7% acetic acid (1 mL/100g) in mice. (**B**) Response latency (s) in hot plate test measured hourly for 4 h after the vehicle, extract (p.o., 200 and 400 mg/kg) or nalbuphine (p.o., 10 mg/kg) administration in mice. Data are presented as mean values ± S.E.M. (n = 5–8). * *p* < 0.05 vs. control values.

The oral treatment with the HCF extract (p.o., 400 mg/kg) 1 h before 0.7% acetic acid injection (1 mL/100 g) showed a significant analgesic effect observed as a decrease in the number of writhes induced by acetic acid injection in mice by 60%. This effect was comparable to that obtained with the mice pre-treated with diclofenac (p.o., 20 mg/kg). The steroidal anti-inflammatory drug, dexamethasone (p.o., 2 mg/kg), showed the strongest analgesic effect; it decreased the writhing number by 72% (Figure 5A).

### 3.7. Effect of the Extract on the Response Latency in Hot Plate Test with Mice

As shown in Figure 5B, mice pre-treated with the HCF extract doses (i.p., 200 and 400 mg/kg) showed longer response latency in a dose-dependent manner when measured at 1, 2, 3 and 4 h after administration. The effect peaked at 3 h after treatment (3-fold of the control). Interestingly, the areas under the response latency-time curve of the 400 mg/kg dose and nalbuphine, the narcotic analgesic used as a reference standard in this experiment, were similar (3705 and 3690 s/min, respectively).

### 3.8. Effect of the Extract on Pyrexia in Mice

Injection of brewer’s yeast in mice raised the rectal body temperature that was measured 18 h after injection (Table 3). Mice treated with the HCF extract (p.o., 200 and 400 mg/kg) showed fast (observed 30 min post-treatment) but brief antipyretic effect. The highest dose (p.o., 600 mg/kg) did not result in any improvement (data not shown). Paracetamol (150 mg/kg) exerted its antipyretic effect starting 1 h following the treatment and was consistent throughout all measurements (Table 3).

### 3.9. Effects of H. campechianum Flowers Extract on Pain and Inflammation in CCI Neuropathic Pain Model

#### 3.9.1. Effect of the Extract on Heat Hyperalgesia and Cold Allodynia

**Figure 6:**
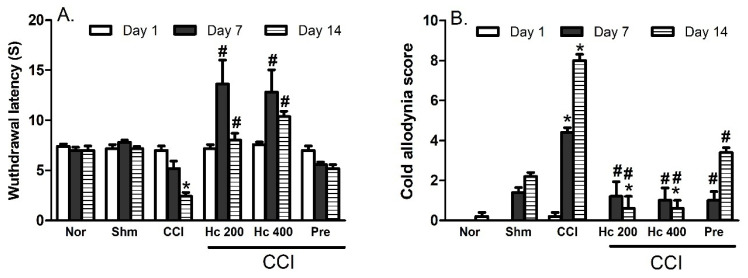
Effects of *H. campechianum* flower extract (200 and 400 mg/kg) on (**A**) heat hyperalgesia (withdrawal latency) and (**B**) cold allodynia responses in rats with neuropathic pain induced by ligation of spinal nerve. Data are presented as mean values ± S.E.M (n = 5). * *p* < 0.05, compared to sham group; # *p* < 0.05, compared to CCI group at the corresponding time points. CCI, chronic constriction injury; Hc, *H. campechianum*; Nor, normal; Pre, pregabalin; Shm, sham.

Rats exposed to CCI showed signs of both heat hyperalgesia and cold allodynia responses represented as time-dependent decrease and increase in the heat response latency time and cold allodynia scores, respectively, when compared to the sham group (Figure 6A,B). The rats treated with the extract (200 and 400 mg/kg) showed prolonged heat response latency, achieving higher values than the control group when measured at days 7 and 14 post-CCI. Interestingly, this response was even higher than that observed with the normal rats, indicating the strong central analgesic effect of the extract. Moreover, the extract-treated group restored the normal cold allodynia responses. Noteworthy, the extract showed superiority over the reference standard, pregabalin, in response to both heat and cold stimuli (Figure 6A,B).

#### 3.9.2. Effect of the Extract on Mechanical Hyperalgesia

**Figure 7:**
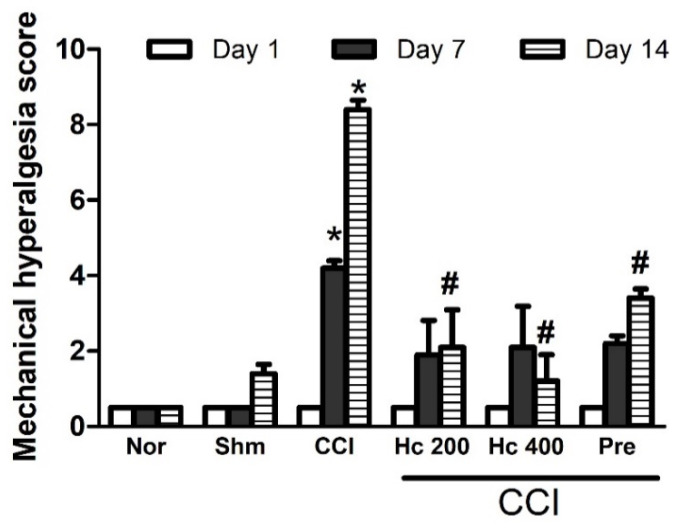
Effect of *H. campechianum* flower extract (p.o., 200 and 400 mg/kg) on the score of mechanical hyperalgesia in rats with neuropathic pain induced by spinal nerve ligation. Withdrawal duration was measured in seconds and ranged from 0.5 s for the brief normal response to 20 s (the cut-off time). Data are presented as mean values ± S.E.M (n = 5). * *p* < 0.05, compared to sham group; # *p* < 0.05, compared to CCI group at the corresponding time points. CCI, chronic constriction injury; Hc, *H. campechianum*; Nor, normal; Pre, pregabalin; Shm, sham.

Mechanical hyperalgesia was significantly increased because of CCI. This was assessed by the pin brick test performed on days 7 and 14 that showed an increase in the withdrawal time of the injured paw compared to the sham group by 4- and 8.4-fold, respectively (Figure 7). Rats treated with the extract (p.o., 200 and 400 mg/kg) showed an attenuated effect, obviously starting at day 7 post-CCI. Interestingly, the higher dose (400 mg/kg) showed a comparable score to the sham group when assessed at day 14 post-CCI. It is notable that the effect of the extract was stronger than that of pregabalin.

#### 3.9.3. Effect of the Extract on Mechanical Dynamic Allodynia

**Figure 8:**
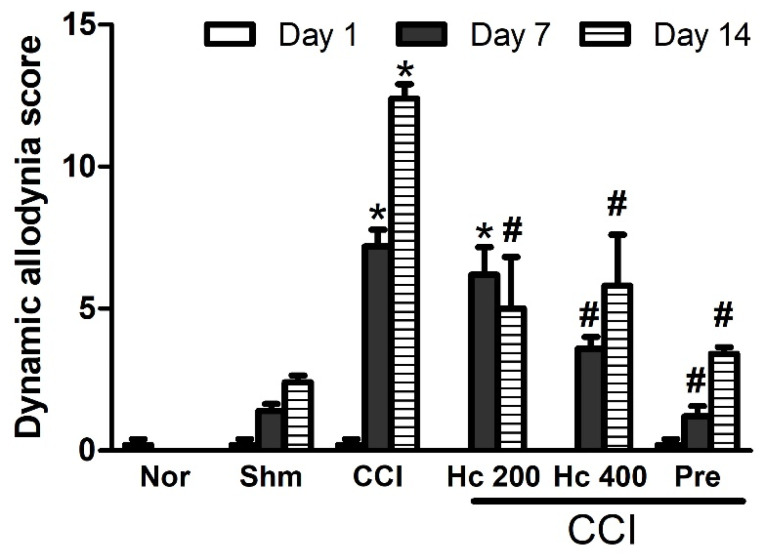
Effect of *H. campechianum* flower extract (p.o., 200 and 400 mg/kg) on mechanical dynamic allodynia in rats with neuropathic pain induced by ligation of spinal nerve. Withdrawals numbers of 15 trials in response to a stimulus induced by paint brush was noted (between 0 and 15). Data are presented as mean values ± S.E.M (n = 5). * *p* < 0.05, compared to the sham group; ^#^ *p* < 0.05, compared to the CCI group at the corresponding time points. CCI, chronic constriction injury; Hc, *H. campechianum*; Nor, normal; Pre, pregabalin; Shm, sham.

Rats exposed to CCI had a significantly (*p* < 0.001) higher dynamic allodynia scores when assessed by the paint brush test on days 7 and 14 following the surgery compared to the sham group (7.2 ± 0.58 for CCI group vs. 1.4 ± 0.25 for sham after 7 days and 12.4 ± 0.51 for CCI vs. 2.4 ± 0.25 for sham after 14 days, respectively). Rats treated with *H. campechianum* flower extract (p.o., 200 and 400 mg/kg) decreased the dynamic allodynia scores in a dose-dependent manner compared to the CCI group. The high dose of the extract exerted a similar effect to that of pregabalin (Figure 8).

#### 3.9.4. Effect of the Extract on CCI-Induced Increase in COX2, LOX and PGE2

**Figure 9:**
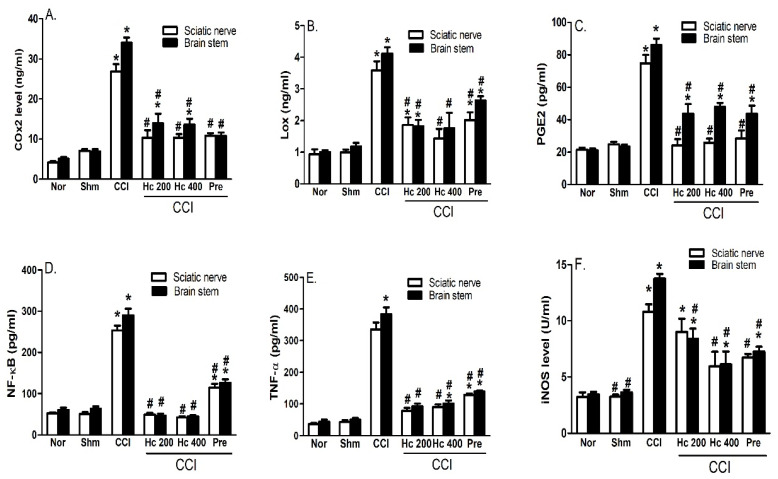
Effect of *H. campechianum* flower extract (p.o., 200 and 400 mg/kg) on CCI-induced increase in COX2 (**A**), LOX (**B**), PGE2 (**C**), NF-κB (**D**), TNF-α (**E**) and iNOS (**F**) levels in the brain stems and sciatic nerves of CCI rats. Values are given as mean ± S.E.M., n = 5 rats/group. * *p* < 0.05, compared to the sham group; # *p* < 0.05, compared to the CCI group. CCI, chronic constriction injury; COX2, cyclooxygenase 2; Hc, *H. campechianum*; iNOS, inducible nitric oxide synthase; LOX, lipoxygenase; NF-κB, nuclear factor kappa B; Nor, normal; PGE2, prostaglandin E2; Pre, pregabalin; TNF- α, tumor necrosis factor alpha; Shm, sham.

Rats exposed to CCI (14 days) showed a significantly (*p* < 0.05) increased COX2 (3.8- and 4.9-fold), LOX (3.6- and 3.5-fold) and PGE2 (3- and 3.7-fold) levels in sciatic nerves and brain stem, respectively, compared to the sham group. This response was attenuated when measured 14 days following treatment with the extract (p.o., 200 and 400 mg/kg) and demonstrated significantly lower sciatic and brain stem COX2 (61% and 60%, respectively), LOX (48–60% and 56–57%, respectively) and PGE2 (65–68% and 44–49%, respectively) levels compared to the control values (Figure 9A–C).

#### 3.9.5. Effect of the Extract on NF-κB, TNF-α and iNOS

Rats exposed to CCI for 14 days showed significantly elevated levels of NFκB (5- and 4.6-fold, respectively), TNF-α (7.7- and 7.8-fold, respectively) and iNOS (3.4- and 3.8-fold, respectively) in sciatic nerves and brain stem compared to rats of the sham group. Brain stems and sciatic nerves obtained from rats treated with the extract (p.o., 200 and 400 mg/kg) for 14 days showed abrogated NFκB levels and attenuated TNF-α (by 64–77%) and iNOS (by 17–56%) levels when compared to the control group rats (Figure 9D–F).

#### 3.9.6. Effect of the Extract on CCI-Induced Oxidative Status

**Figure 10:**
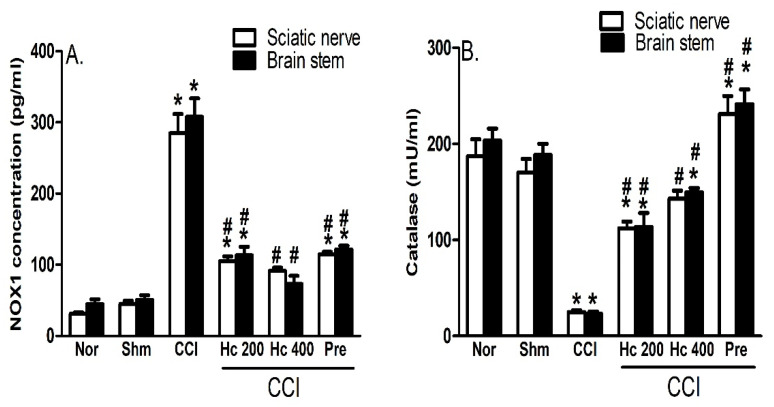
Effect of *H. campechianum* flower extract (p.o., 200 and 400 mg/kg) on CCI-induced rise in NOX1 level (**A**) and catalase activity (**B**) in the brain stems and sciatic nerves of CCI rats. Values are given as mean ± S.E.M., n = 5 rats/group. * *p* < 0.05, compared to the sham group; # *p* < 0.05, compared to the CCI group. CCI, chronic constriction injury; Hc, *H. campechianum*; Nor, normal; NOX1, NADPH oxidase; Pre, pregabalin; Shm, sham.

As shown in Figure 10, brain stems and sciatic nerves of CCI rats showed a higher oxidative status represented by significantly (*p* ˂ 0.0001) elevated levels of NADPH oxidase (NOX1) and lower levels of the antioxidant enzyme catalase compared to the sham group, whereas rats treated with the extract (p.o., 200 and 400 mg/kg) for 14 days showed an improved oxidative status represented by dose-dependent decreased NOX1 activity and an increased catalase activity measured in both preparations and compared to the values obtained from the sham rats (Figure 10A,B).

### 3.10. Effect of the Extract on Brain Stem p53 Immunohistochemistry

**Figure 11:**
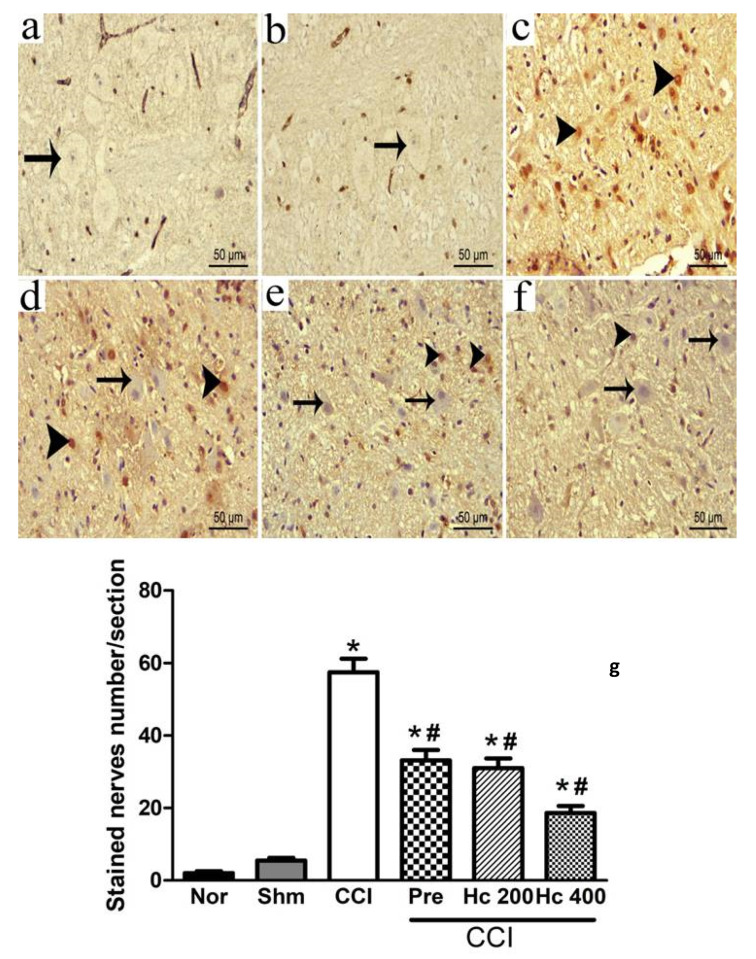
Representative photomicrographs revealing the apoptotic neurons expression via p53 (**a**–**f**) immunostaining brain stem sections in normal (**a**), sham (**b**), CCI (**c**), pregabalin (**d**) *H. campechianum* flower extract (p.o., 200 mg/kg) (**e**); and *H. campechianum* flower extract (p.o., 400 mg/kg) (**f**). Head arrow indicates dark brown staining nuclei of immunopositive neurons and the arrow indicates the negative neurons. Bar chart (**g**) shows the changes in the p53 positive neurons’ number with dark brown nuclei in the brain stem sections from all experimental groups. Counting the immunopositive neurons was conducted in 3 non-overlapping fields from 100% magnification (overall 6 animals/group). Data are presented as mean values ± S.E.M (n = 5–7). * *p* < 0.05 vs. normal values, ^#^ *p*< 0.05 vs. CCI. Scale bar 50 μm. CCI, chronic constriction injury; Hc, *H. campechianum*; Nor, normal; Pre, pregabalin; Shm, sham.

Brain stem tissues were stained with anti- p53 to investigate the presence of apoptotic neurons. The p53-positive neurons were detected by showing dark brown nuclei. They were negative in the control group but occasionally noticed in the sham group, which showed no significant difference relative to the control group. On the contrary, the immunopositive cells were apparently detected in the CCI group, which showed a significant up-regulation of p53 levels relative to the control group. Immunopositive neurons were downregulated in either group treated with pregabalin or a lower extract dose (p.o., 200 mg/kg). However, the immunopositive neuron numbers still significantly differed relative to the control group. The group treated with the higher extract dose (p.o., 400 mg/kg) showed a stronger reduction in the positive cell numbers when compared to the lower dose (Figure 11a–g).

### 3.11. Effect of the Extract on Brain Stem GFAP Immunohistochemistry

**Figure 12:**
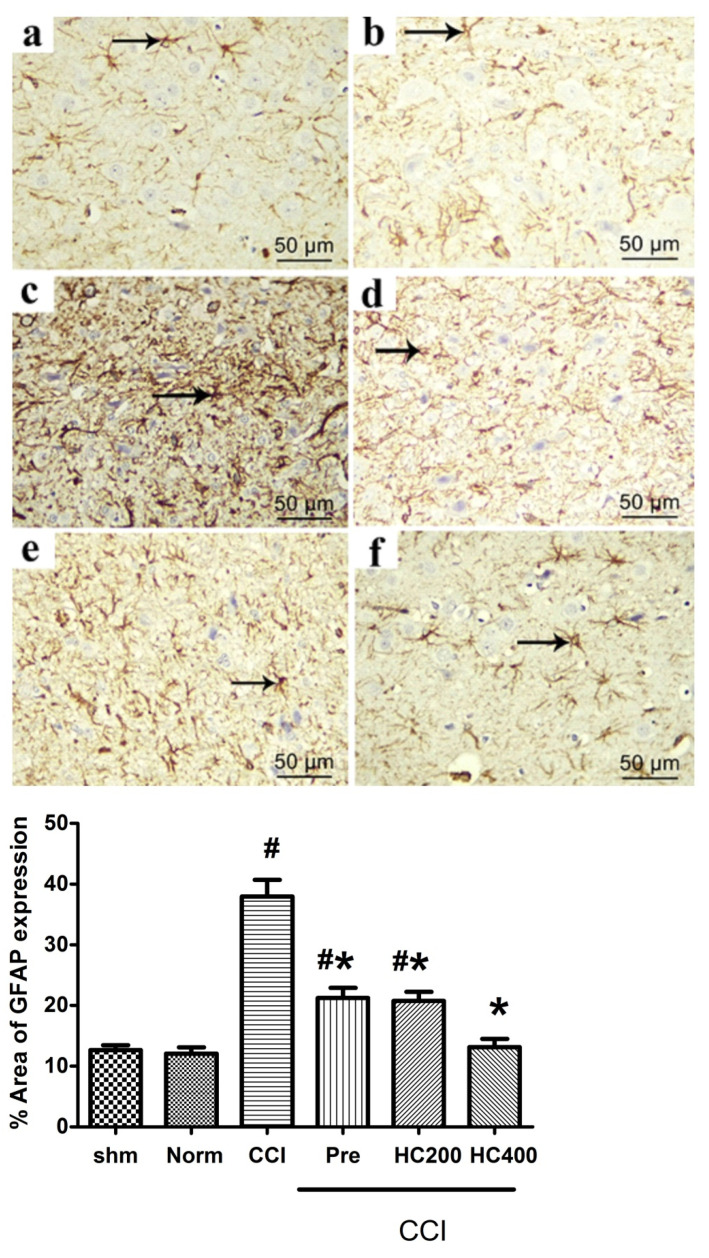
Photomicrographs of the brain stem of the normal (**a**), sham (**b**), control (CCI) group (**c**), pregabalin group (**d**), *H. campechianum* flower extract (p.o., 200 mg/kg) (**e**); and *H. campechianum* flower extract (p.o., 400 mg/kg) (**f**) groups. Bar chart demonstrating the area % of GFAP positive astrocyte in the brain stem of the different experimental groups. Statistical analysis was carried out using one-way ANOVA, followed by Tukey’s post hoc test. Values are represented as the mean ± SEM (n = 8). # significant difference compared to the control group, *p* < 0.05. * Significant difference compared to the diseased group, *p* < 0.05.

The brain stem was immunohistochemically stained with the anti-GFAP antibody to elucidate the response of astrocytes to the neural damage in the different studied groups. Normal and sham groups showed GFAP positive staining in the cytoplasm of astrocytes and their processes. They appeared small and few with short, thin few processes (Figure 12a). However, in the CCI group, there was abundant GFAP positive staining of the cytoplasm and the processes of astrocytes. They were apparently increased in number and appeared larger with multiple long thick processes (Figure 12b,c). In pregabalin and *H. campechianum* flower extract (p.o., 200 mg/kg) groups showing astrocytes with thin, short ramified processes (Figure 12d,e). On the contrary, in the *H. campechianum* flower extract (p.o., 400 mg/kg) group, the astrocyte was small and few in numbers with short, thin few processes (Figure 12f). Statistical analysis of area % of GFAP reactivity revealed a significant increase in the area % of reactivity in the CCI group compared to the normal and sham groups. In pregabalin and *H. campechianum* flower extract (p.o., 200 mg/kg) groups, there was a significant decrease in area % of GFAP reactivity compared to the CCI group but still showed a significant difference from the control group. On the other hand, the *H. campechianum* flower extract (p.o., 400 mg/kg) group revealed a significant decrease in the area % of GFAP reactivity, which reaches the normal value.

### 3.12. Effect of the Extract on Sciatic Nerves

#### 3.12.1. Effect on Histological Structure

**Figure 13:**
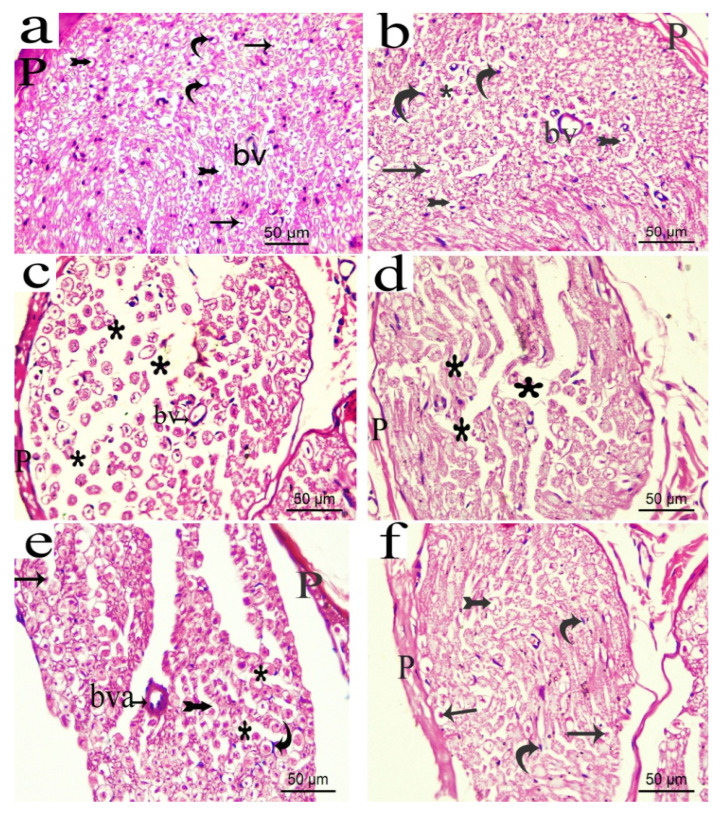
Photomicrograph (H&E × 400) of transverse sciatic nerve sections from: normal (**a**); sham (**b**); CCI (**c**); pregabalin (**d**); *H. campechianum* flower extract (200 mg/kg) (**e**); *H.* *campechianum* flower extract (400 mg/kg) (**f**) rats. Rats received different treatments for 14 days after which the animals were sacrificed and tissues were collected. Arrow, bifid arrow, curved arrow and asterisks illustrate axoplasm, area of dissolved myelin, Schwann cells nuclei and wide separation between the nerve fibres, respectively. (bv) blood vessel, (bva) dilated blood vessel, (P) perineurium. Scale bar, 50 μm x 400.

H&E staining of the transverse section of rat sciatic nerve from the normal or the sham groups showed nerve fascicles with closely packed nerve fibres surrounded by an intact sheath of connective tissue (perineurium). Myelinated nerve fibres were formed by axoplasm surrounded by an unstained area of dissolved myelin with Schwann cells nuclei, which appeared in-between the nerve fibres. An occasional endoneurial blood vessel could be seen. In the CCI group, transverse sciatic nerve sections showed disorganization of nerve fascicles with most of the nerve fibres widely separated from each other and the overlying perineurium. Moreover, thick endoneurial blood vessel was also observed. In CCI groups treated with pregabalin or the lower extract’s dose (p.o., 200 mg/kg), the nerve fascicle restored most of its normal appearance, but the nerve fibres were still widely separated. However, sections obtained from sciatic nerves of the rats treated with the higher extract’s dose (p.o., 400 mg/kg), showed apparently normal nerve fascicles formed mostly of myelinated nerve fibres and Schwann cells nuclei in endoneurial spaces with a slight separation between the nerve fibres (Figure 13a–f).

**Figure 14:**
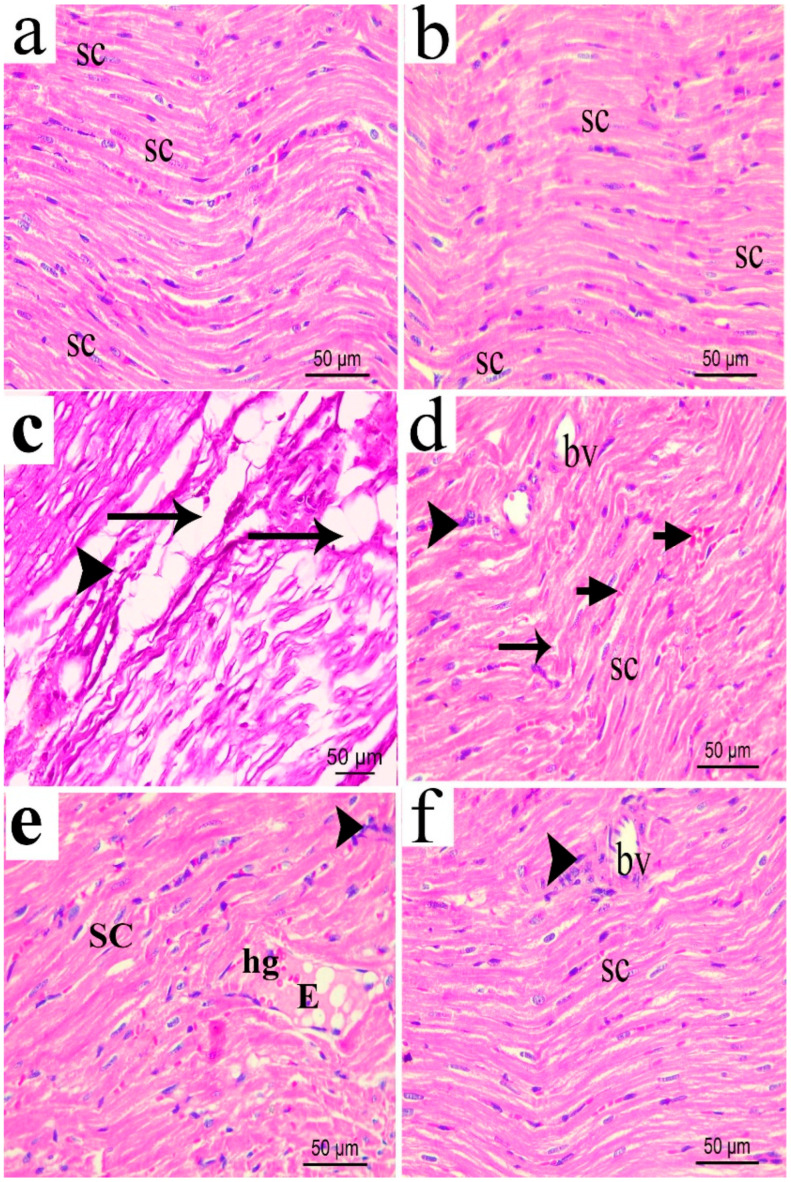
Photomicrograph (H&E × 400) of longitudinal sciatic nerve sections from normal (**a**); sham (**b**); CCI (**c**); pregabalin (**d**); *H. campechianum* flower extract (200 mg/kg); and (**e**) *H. campechianum* flower extract (400 mg/kg) (**f**) rats. Rats were subjected to different treatments for 14 days, after which the animals were sacrificed, and tissues were collected. Arrow and arrowheads illustrate myelin sheet degeneration and mononuclear infiltrating cells, respectively. bv, blood vessel; E, exudate; hg, hemorrhage; SC, Schwan cell nuclei. Scale bar, 50 μm.

Similarly, longitudinal sections of the normal and the sham sciatic nerves (Figure 14a–f) showed normal architecture and the absence of infiltrating cells. On the other hand, sections of sciatic nerves exposed to CCI revealed several areas of edema and degraded myelin sheets and some infiltrating mononuclear cells. Hyalinization and hemorrhage in between the fibres could be also observed. CCI rats treated with either pregabalin or low extract dose (200 mg/kg) showed more organized nerve fibres less edematous reaction, fewer cellular infiltrations, less extravagated hemorrhage and hyalinisation between the fibres compared to the vehicle-treated CCI sections. The extract’s higher dose (p.o., 400 mg/kg) restored most of the normal nerve architecture with only a few infiltrating cells observed.

#### 3.12.2. Effect of the Extract on Sciatic Nerve Integrity

**Figure 15:**
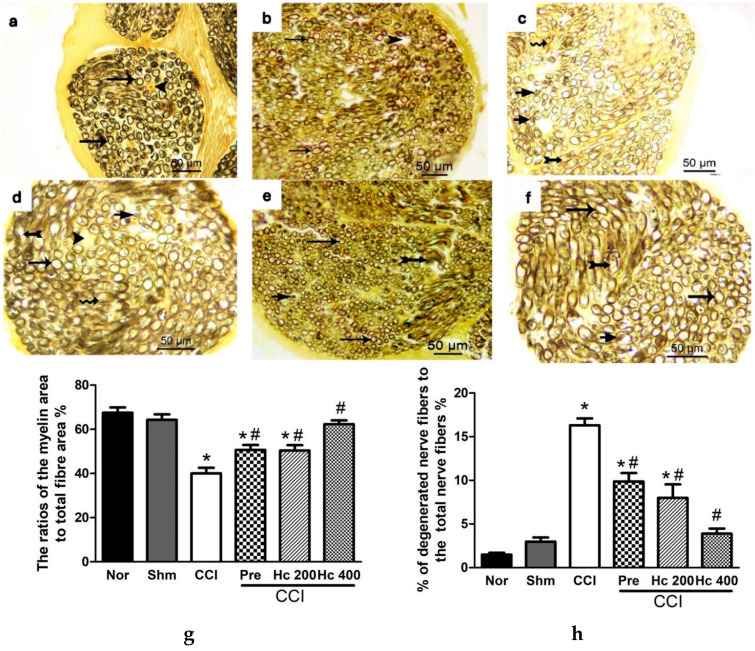
Photomicrographs of sciatic nerve transverse sections stained with osmic acid in (**a**) normal group; (**b**) sham group; (**c**) CCI group; (**d**) pregabalin group; (**e**) *H. campechianum* flower extract (p.o., 200 mg/kg) group; and (**f**) *H. campechianum* flower extract (p.o., 400 mg/kg) group. Arrow, arrowhead, bifid arrow, short arrow, and wavy arrow illustrate the normal myelin sheath concentric lamellar structure, unmyelinated nerve fibres, compromised normal myelin sheath concentric lamellar structure, and invagination of the myelin sheath into the axon and axonal swelling, respectively. Lower panel represents the ratio of myelin sheath to the total nerve area (**g**) and the ratio of degenerated nerve fibres to the total number of nerve fibres (**h**). Data are presented as mean values ± S.E.M. (n = 5–7). * *p* < 0.05, compared to the sham group; ^#^ *p* < 0.05, compared to the CCI group. Scale bar, 50 μm × 400. CCI, chronic constriction injury; Hc, *H. campechianum*; Nor, normal; Pre, pregabalin; Shm, sham.

Sciatic nerve sections stained with osmic acid revealed that the ratios of the myelinated area to the total nerve fibre area were similar in both normal and sham groups (67.35 ± 6.66% and 64.29 ± 2.46%, respectively). In contrast, this ratio was significantly decreased in sciatic nerve sections obtained from the CCI group (40.06 ± 2.35%), indicating myelin sheath degeneration. Interestingly, the extract in the low dose (200 mg/kg) showed partial restoration of this ratio to reach 50.64 ± 2.25%, an effect that was comparable to that of pregabalin (Figure 15a–h). The higher extract’s dose (p.o., 400 mg/kg) restored completely the myelin sheath integrity indicated by the ratio of the myelin area to the nerve fibre area, which increased to a value that was not significantly different from the sham value (62.31 ± 1.76%) (Figure 15a–h). Moreover, the percentage of the degenerated nerve fibres to the total nerve fibres count was significantly higher in the CCI group relative to the normal and sham groups. CCI rats treated with the lower extract’s dose (p.o., 200 mg/kg) or pregabalin showed a significant reduction in the number of degenerated nerve fibres relative to the vehicle-treated CCI rats. Notably, the higher extract dose (p.o., 400 mg/kg), in addition, restored the normal nerve architecture and integrity (Figure 15a–h).

### 3.13. Investigation of Chronic Ulcerogenic Activity of H. campechianum Flower Extract

#### 3.13.1. Macroscopic Examination of Rat Stomachs

As shown in Table 4, stomachs obtained from CCI rats had a small ulcer index (2.75) and showed red coloration in 40% of the examined stomachs. Macroscopic examination of the stomachs obtained from rats treated for 14 days with the HCF extract showed an increased ulcer index (9.5) with some (75%) of the stomachs having either red coloration or minute number of spot ulcers. Interestingly, the previous mild side effect on the stomach did not aggravate upon increasing the dose to 400 mg/kg but rather, a lower ulcer index (6.31), a smaller number of animals with ulcer (50%) and a lower severity score (0.56) were observed compared to the lower dose. The ulcer indices of the extract’s two doses were higher than that of the selective COX2 inhibitor, celecoxib, but much less than that of the non-selective inhibitor, indomethacin, which induced severe ulceration in stomachs with very high ulcer index (24.48; Table 4). These results support our data obtained from the in vitro enzyme inhibition assays that showed the extract’s selectivity to COX2 (SI = 16.9).

#### 3.13.2. Effect of the Extract on Stomach

**Figure 16:**
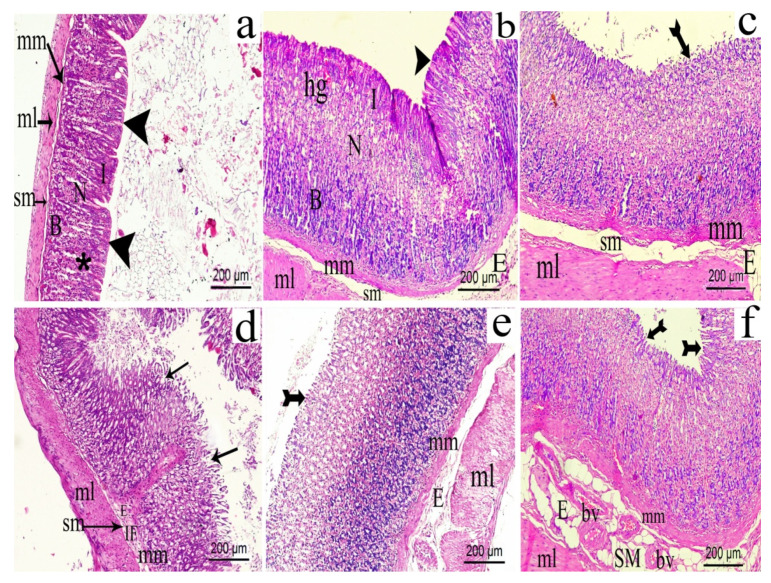
Photomicrograph of gastric mucosa of rat stomach sections from: (**a**) normal group; (**b**) celecoxib group; (**c**) CCI group; (**d**) indomethacin group; (**e**) *H. campechianum* flower extract (p.o., 200 mg/kg) group; and (**f**) *H. campechianum* flower extract (p.o., 400 mg/kg) group. Head arrow, bifid arrow, arrow and asterisk illustrate intact gastric epithelium, slight erosion in gastric epithelium, shedding and ulcer in gastric epithelium and gastric pit, respectively. Basic region (B); blood vessel (bv); edema (E); hemorrhage (hg); infiltration (IF); Isthmus (I); mucosa (M); muscular layer (ml); muscularis mucosa (mm); neck (N); and submucosa (sm). Scale bar, 200 μm × 100.

Rat stomach sections stained with H&E showed normal architecture of stomach, both in the normal and sham groups. Gastric mucosa revealed normal layers of epithelium, lamina propria containing gastric glands and muscularis mucosa. The fundic glands revealed regular structure formed of isthmus, neck and base regions and opened to the surface via narrow gastric pits. The epithelium was found intact with no evidence of inflammatory cells infiltration or edema in submucosal layer (Figure 16a). On the other hand, stomach sections of the CCI group showed a slight hemorrhage in the lamina propria with slight edema in the submucosal layer (Figure 16b). Interestingly, sections obtained from celecoxib group (Figure 16c) and the extract (200 mg/kg) group (Figure 16e) showed similar histological changes represented as slight erosion in the epithelium, increased muscularis mucosa thickness and submucosal edema, while the indomethacin treated stomachs showed well-developed gastric ulcers and erosion with inflammatory cells infiltration (Figure 16d). The higher extract’s dose (p.o., 400 mg/kg) resulted in significant sub-mucosal edema with congested submucosal blood vessels (Figure 16f).

### 3.14. Effect of the Extract on Brain Stem

**Figure 17:**
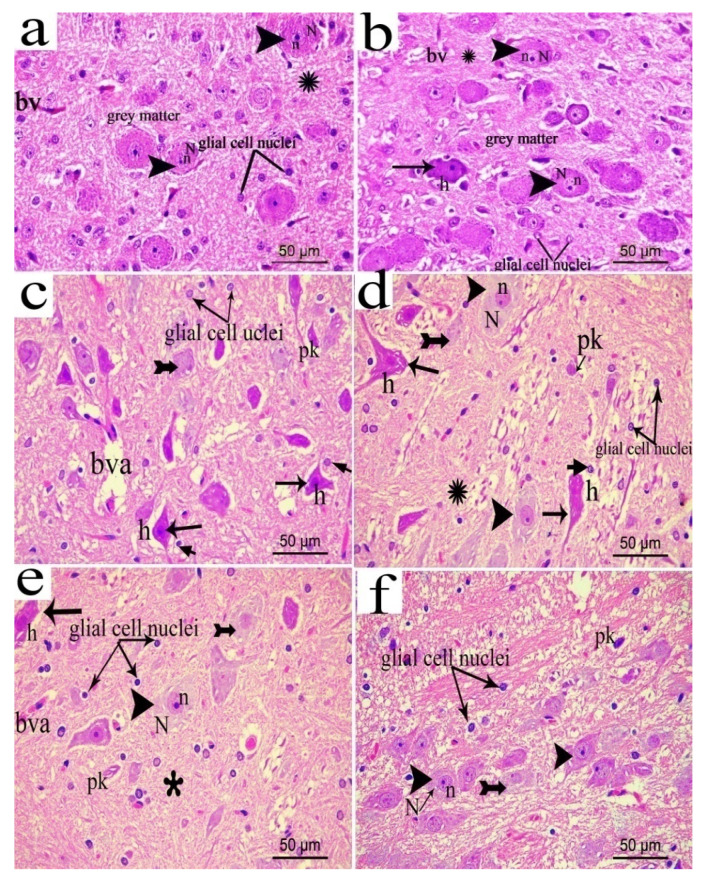
Representative photograph in sections of brain stem from: (**a**) normal; (**b**) sham; (**c**) CCI; (**d**) pregabalin; (**e**) *H. campechianum* flower extract (p.o., 200 mg/kg); and (**f**) *H. campechianum* flower extract (p.o., 400 mg/kg) group. Arrowhead, arrow, bifid arrow, short arrow and asterisk (*) illustrate normal neurons, affected neurons with dark stained cytoplasm and nuclei, neurons with rarified lightly stained nucleus, perineural glial cell nuclei and neuropil, respectively. bv, normal blood vessel; bva, dilated or congested blood vessel; h, vacuolation and pk, pyknotic nuclei and glial cells nuclei; N, nissl granule; n, nuclei. Scale bar, 50 μm × 400.

H&E stained brain stem sections of the normal group revealed normal neurons in the gray matter with vesicular nuclei and basophilic cytoplasm contained Nissl granule prominent nuclei. The acidophilic neutrophil contained normal glial cells nuclei and normal blood vessels with narrow perivascular spaces (bv). In the sham group, few affected neurons with dark stained nuclei and shrunken cytoplasm surrounded by pericellular halo were seen. In contrast, the CCI group, revealed some neurons with large rarified lightly stained nuclei, while other neurons were degenerated, where they had either shrunken, elongated, irregular, or deeply stained nuclei with extensively vacuolated cytoplasm or pyknotic, small and deeply stained nuclei (indicative of apoptotic reactions). Oligodendrocytes were seen in close relation to some degenerated neurons. In addition, the neuropil was vacuolated and contained dilated congested blood vessels. In the pregabalin group, most neurons were affected, and few neurons were normal. Administration of the low extract’s dose (200 mg/kg) revealed partially counteracted degenerative changes of neurons where some neurons were normal, and some were affected with less vacuolated neuropil and a normal blood vessel. Meanwhile, administration of the higher extract’s dose (p.o., 400 mg/kg) restored intact neurons where most of them were normal and only a few showed pyknotic, small and deeply stained nuclei or large rarified lightly stained nuclei (Figure 17a–f).

### 3.15. Effect of H. campechianum Flower Extract on the Kidney

**Figure 18:**
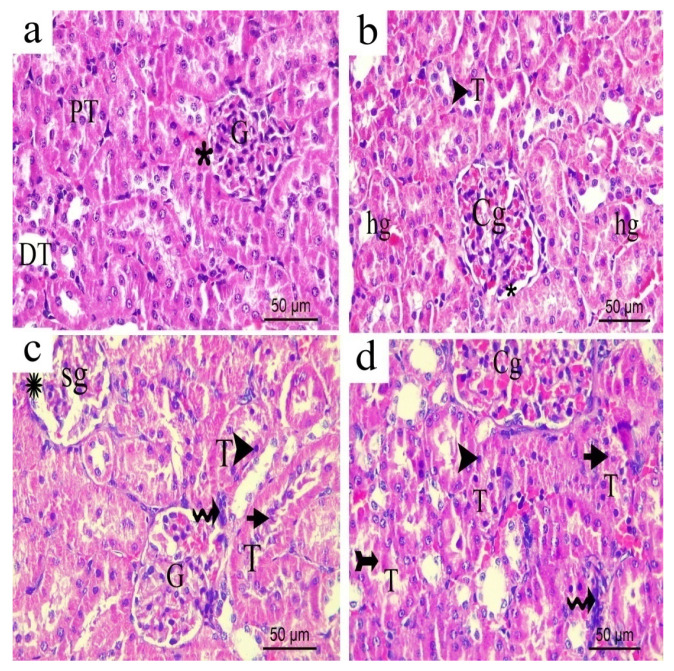
Representative photograph of renal cortex sections of the different groups. (**a**) sham; (**b**) CCI; (**c**) *H. campechianum* flower extract (p.o., 200 mg/kg); (**d**) *H. campechianum* flower extract (p.o., 400 mg/kg). Asterisk, arrow and arrowhead illustrate bowman space the dark, stained nuclei and infiltrating cells, respectively. C, peritubular capillaries; Cg, congested glomeruli; DT, distal convoluted tubule; E, exfoliated cells; G, glomerulus; PT, proximal convoluted tubule; T, affected tubule. Scale bar, 50 μm × 400.

The H&E stained renal cortex sections from the sham or normal groups exhibited normal renal corpuscles with glomerular capillaries tuft and normal bowman space. Normal proximal and distal convoluted tubules were observed (Figure 18a). Kidneys of rats exposed to CCI showed few tubules that revealed dark stained apoptotic nuclei with congested glomerulus along with some extravasation of hemorrhage between the renal tubules (Figure 18b). Noteworthy, kidneys obtained from CCI rats treated with the extract (200 mg/kg) for 14 days, showed similar histopathological changes compared to the vehicle-treated CCI rats except for the appearance of some tubules with exfoliated cells in their lumen along with slight periglomerular cellular infiltrations (Figure 18c). Doubling the extract’s dose resulted in more renal side effects represented by the appearance of congested glomerulus with hemorrhagic extravasation in- between renal tubules (Figure 18d).

### 3.16. Effect of H. campechianum Flower Extract on the Liver

**Figure 19:**
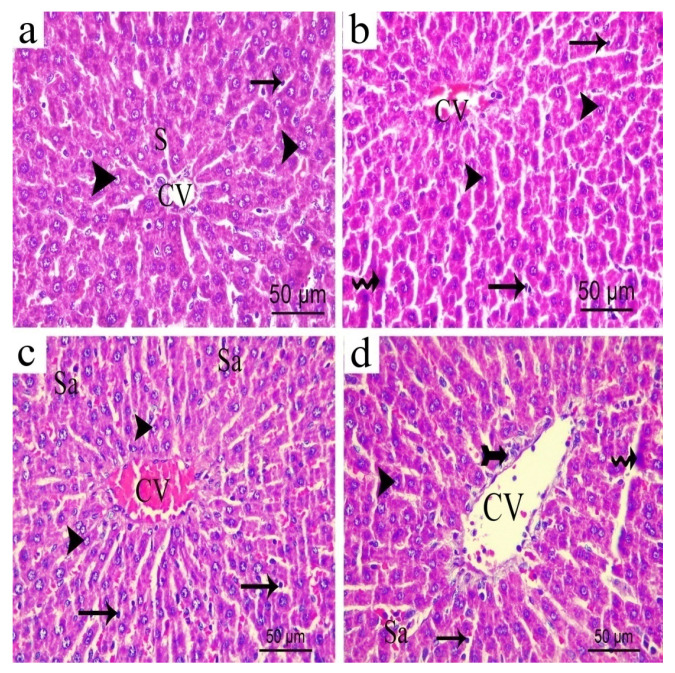
Representative photograph of liver sections from: (**a**) normal; (**b**) CCI; (**c**) *H. campechianum* flower extract (p.o., 200 mg/kg); (**d**) *H. campechianum* flower extract (p.o., 400 mg/kg). Head arrow, arrow, wavy arrow and bifid arrow illustrate normal hepatocytes, Kupffer cell, hepatocytes with dark stained nuclei and cellular infiltrations, respectively. CV, the central vein; S, normal blood sinusoids; sa, dilated or congested sinusoids. Scale bar, 50 μm × 400.

H&E stained liver sections of either the sham or the normal group revealed a normal architecture of the liver hepatocytes, having vesicular nuclei and basophilic cytoplasm. They were radiating from the normal central vein and separated by normal sinusoids with Kupffer cells (Figure 19a). Liver sections obtained from rats exposed to CCI, on the other hand, revealed slightly dilated and congested central vein with slightly dilated sinusoids. Few hepatocytes with dark stained nuclei indicating apoptotic reaction (Figure 19b) were observed. Meanwhile, CCI rats group treated with the lower extract’s dose (p.o., 200 mg/kg) showed normal architecture of the liver hepatocytes that were radiating from slightly dilated and congested central vein with the occurrence of some dilated congested sinusoids and a significant rise in Kupffer cells number (Figure 19c). The higher extract dose (p.o., 400 mg/kg) resulted in a more dilated central vein with slight cellular infiltrations around it and affected a few hepatocytes to appear with dark stained apoptotic nuclei and dilated congested sinusoids (Figure 19d).

### 3.17. Molecular Docking

The docked compounds from the extract showed appreciable binding affinity to the p53 DNA binding domain indicated by low scoring function values, which were close to or even better than that of the reference inhibitor, bispicen. Table 5 lists the scoring functions and the amino acid interactions of the docked compounds.

**Figure 20:**
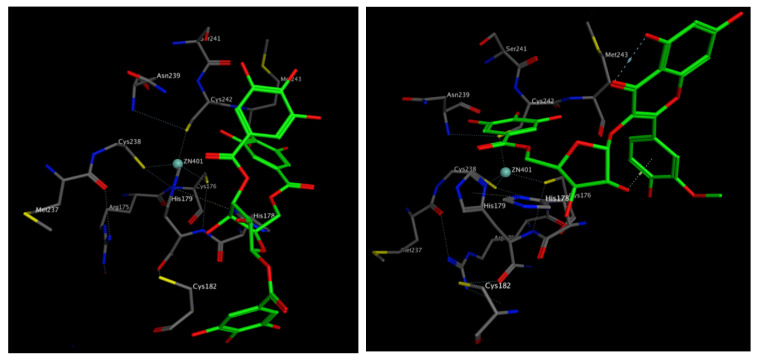
Amino acid interactions with trigalloyl glucose (**left**) and isorhamnetingalloyl- pentoside (**right**) in the DNA binding domain of p53.

Overexpression of p53 in neuronal cells is significantly linked to neuropathic pain and neurons degeneration [34]. Synthetic p53 inhibitors were reported to protect against neuron death in neurodegenerative disorders by chelating the Zn atom in the DNA binding domain [35]. Four amino acids are involved in coordinate interactions with the Zn atom, namely, Cys 176, His 179, Cys 238 and Cys 242 [36]. The docked compounds showed one or more interaction with some of these amino acid residues besides other polar and non-polar interactions in the binding site (Figure 20). Such interactions could lead to disrupting the zinc finger domain geometry, giving an inactive conformation of the protein.

## 4. Discussion

The present study aims to characterize the chemical composition and investigate the potential of *H. campechianum* flowers extract to be used as treatment modality for acute and chronic models of inflammation and pain. The major findings of this study were as follows. (1) the extract has 9 phenolic and 6 flavonoid compounds. (2) *In vitro* study showed that the extract has a strong antioxidant effect and inhibits COX1, COX2 and LOX with selectivity towards COX2. (3) *In vivo* study showed that the extract attenuates carrageenan-induced paw edema, carrageenan-induced leukocyte migration into peritoneal activity and acetic acid-induced vascular permeability and possesses both central and peripheral antinociceptive effects and brief antipyretic effects. (4) Furthermore, it improved chronic neuropathic pain behavior after thermal and mechanical stimuli-induced hyperalgesia and allodynia in a rat CCI model. (5) The proinflammatory cytokines such as TNF-α, NF-κB and PGE2 decreased after extract administration in the sciatic nerve and brain stem of rats in the CCI model. (6) *H. campechianum* flowers extract treatment downregulated the expression of iNOS, COX2, and LOX in the sciatic nerve and brain stem of rats following CCI. (7) It also improved oxidative status via reduction of NOX1 activity and increasing catalase activity in the sciatic nerve and brain stem of rats following CCI. (8) The extract attenuated leukocyte infiltration and structural derangement, restored astrocyte normal appearance and reduced apoptotic marker P53 in both sciatic nerve and brain stem of rats subjected to CCI, which is confirmed by molecular docking of the extract compounds. (9) The extract has minor adverse effects on stomach, liver and kidney.

LC–MS/MS analysis of the extract resulted in the identification of 9 phenolic and 6 flavonoid compounds. Phenolics and flavonoids are characterised by their anti-oxidant and anti-inflammatory properties [37]. In the current study, the biological investigation of *H. campechianum* flower extract revealed its significance as anti-inflammatory, analgesic, antipyretic and antioxidant agent. As for the anti-inflammatory activity, the extract exerts its effect by targeting several inflammatory mediators, including COX1, COX2, and LOX, with a selectivity towards COX2 and by its ability to decrease the migrating leukocytes and vascular permeability leading to less accumulation of liquids at the site of inflammation.

Damage to the central or peripheral nervous system can cause chronic neuropathic pain, which is a prevalent and debilitating condition. Chronic constriction of the sciatic nerve represents one of the best models of chronic neuropathic pain that mimics peripheral nerve injury [38]. It is characterised by allodynia and hyperalgesia. The current treatment strategies used in clinical practices are non-steroidal anti-inflammatory drugs, opiates, tricyclic antidepressants, and anticonvulsant drugs. However, they have either limited efficacy or unacceptable side effects [39]. The present study showed that following CCI of the sciatic nerve, leukocyte infiltration occurs and resident macrophages and Schwann cells are activated. These lead to increased levels of proinflammatory cytokines, particularly NF-κB. The increased level of NF-κB was associated with increased levels of inflammatory mediators such as TNF-α and inflammatory enzymes like COX-1, COX2, LOX, iNOS and NOX1. These lead to an increase in the levels of PGE2, leukotrienes, NO, increased oxidative stress and increased P53 both in the sciatic nerve and brain stem. These changes cause inflammation, destruction of the myelin sheath and increased neuronal apoptosis. Furthermore, it results in pain, hyperalgesia and allodynia. Following a sciatic nerve CCI, free radicals are released and lipid peroxidation rate increases [40]. The increased generation of reactive oxygen species (ROS) not only initiates but also maintains both peripheral and central sensitivity, which result in the hyperalgesic symptoms of neuropathic pain [41]. Treatment with antioxidants has been reported to significantly alleviate thermal hyperalgesia in rats with neuropathic pain. Our results support the findings of Leyva-López et al. and Yahfoufi et al. [42,43] who reported the immunomodulatory and anti-inflammatory properties of polyphenolics and flavonoids, the main components of the HCF extract. In agreement with Yahfoufi et al. [43], the antioxidant capability of the HCF extract can be explained on the basis of increasing the antioxidant catalase enzyme and decreasing NOX1, probably because of its high polyphenolic content. The antioxidant activity of the extract explains its ability to improve both mechanical and thermal hyperalgesia and allodynia. The extract decreased the oxidant enzymes iNOS and NOX1 and increased the antioxidant catalase enzyme, both peripherally in the sciatic nerve and centrally in the brain stem that holds a pain circuit.

Another mechanism involved in chronic neuropathic pain is inflammation. Directly after nerve injury, Schwann cells and local macrophages initiate an immune response, releasing some pro-inflammatory cytokines and algesic mediators in the CNS, such as TNF-α, and other pro-inflammatory cytokines, leading to propagating the inflammatory response over time, which further aggravates central sensitization, hyperalgesia, and allodynia [44]. Previous studies reported elevated messenger RNA (mRNA) and protein expressions of the cytokines IL-1 and TNF-α in injured mice sciatic nerve. Both nerve function and nociceptive sensitivity were potentiated in IL-1β-, TNF-α-, and IL-1β/TNF-knockout mice after sciatic nerve injury [45]. It is reported that ectopic discharges are produced by injured nerve fibres and their dorsal root ganglion (DRG) cells. These ectopic discharges enter the spinal cord and sensitise spinal dorsal horn neurons, thus activate the spinothalamic tract and produced both allodynia and hyperalgesia. Previous studies showed that TNF-α level is increased in dorsal root ganglia following peripheral nerve injury and contributes to apoptosis and central sensitisation [46]. However, little is known about TNF- α expression in the brain stem. The present study showed that CCI was also associated with increased pro-inflammatory cytokines such as TNF-α and NF-κB and increased PGE2 synthesis both in the sciatic nerve and in the brain stem after 14 days of CCI. In our study, we tried to overcome the limitation of Kim et al. [46], who measured TNF-α level after 7 days of peripheral nerve injury. Our study showed that increases in TNF-α and apoptosis affect both initiation and the maintenance of the pain behaviors following CCI of the sciatic nerve. The extract comprises the advantages of decreasing these inflammatory mediators centrally in the brain stem and peripherally in an inflamed sciatic nerve. Previous work showed the importance of phenolics and flavonoids as COX, LOX and PGE inhibitors [46,47,48,49].

The histopathological examination of the injured sciatic nerve showed disorganization of nerve fascicles with most of the nerve fibres widely separated from each other and from the overlying perineurium with hyalinisation and haemorrhage in between the fibres. These results were confirmed by osmic stain of the sciatic nerve. These findings are in line with previous studies [50,51]. The HCF extract counteracted these degenerative changes where the sciatic revealed the normal nerve architecture and integrity after HCF administration. The histopathological examination of the brain stem of the CCI group supported our results, represented by degenerative neurons that had dark stained cytoplasm and nuclei and surrounded by pericellular vaculation. There was a significant increase in the number of p53 immunopositive neurons, which is the biomarker of apoptosis. This is in accordance with previous reports that revealed the localization of p53 immunoreactivity in most of the dorsal root ganglion neurons of rats with chronic constriction of the sciatic nerve

In order to investigate the changes of astrocytes in the brain stem following CCI of the sciatic nerve, we measured the changes in Glial fibrillary acidic protein (GFAP) a protein expressed in astrocytes in CNS. The area percentage (%) of GFAP immunoreactivity was significantly increased in the CCI group as compared with the control group. This is in accordance with Nishihara et al. [52] who reported that the activation of astrocytes, characterized by enhanced GFAP-immunoreactivity, was observed in both the anterior horn and posterior horn of spinal cord in a CCI model. The increase in GFAP expression has been documented as a biomarker of neuronal toxicity and injury, which stimulates the astrocytes’ proliferation and hypertrophy with subsequent increase in the synthesis of GFAP, leading to vigorous astrogliosis which is a compensatory neuro-protective process [53]. The HCF extract counteracted these deleterious changes of the neurons.

The ability of an injured sciatic nerve to retain its normal appearance, and the low expression of the neurodegenerative marker, p53, in the brain stem as a response to HCF extract, enhances the importance of the extract as an anti-inflammatory agent both on the peripheral and central levels. The histopathological examination of the brain stem supported our results. The HCF extract counteracted the degenerative changes of the neurons, which showed to retain their normal structure with vesicular nuclei and basophilic cytoplasm containing Nissl granule. These results were confirmed by osmic stain of the sciatic nerve, which revealed the normal nerve architecture and integrity after HCF administration.

Concerning the adverse effects of the extract, we investigated the effects of 14 days administration on stomach, kidney and liver in a CCI model. Ulcerogenic examination of HCF extract effects revealed its mild effect on the stomach compared to the available anti-inflammatory drugs like indomethacin, which makes the extract a better natural alternative. A treatment limitation, however, was associated with the extract’s higher doses, which exhibited an adverse effect on kidney and liver, as revealed by mild structural changes.

The present study has some limitations. The first limitation of the present study is that we did not measure IL-1β or anti-inflammatory cytokines such as IL-10. The second limitation is that we did not measure inflammatory cytokines or oxidative stress markers in either dorsal root ganglia or spinal cord. Justification for these limitations is that many previous studies have investigated them previously with little attention paid to the brain stem, which plays a crucial role in the pain circuit

## 5. Conclusions

The study explores the pharmacological properties of a flower extract from *H. campechianum* on both acute and chronic models of pain and inflammation. The extract demonstrated both peripheral and central analgesic and anti-inflammatory properties in addition to antipyretic effects mediated through targeting COX, LOX and PGE. The extract ameliorated the neuropathic pain via inhibiting the NF-κB/TNF-α/NOX/iNOS signalling pathway in a rat model of chronic constriction injury in addition to retaining the integrity of the sciatic nerve. It showed antioxidant potential evidenced by elevating the catalase enzyme and reducing the NOX1 production. These diverse biological activities might be attributed to the presence of 15 secondary metabolites, mainly gallotannins and flavonoids. The *H. campechianum* flower extract could be a promising candidate for treating inflammation and neuropathic pain.

## Figures and Tables

**Table 1:** List of identified secondary metabolites from *H. campechianum* flowers using LC–MS/MS analysis.

No.	RT	M-H *m/z*	MS/MS	Tentitatively identified compounds	Ref.
1	2.24	169	125	Gallic acid	[25]
2	11.72	321	125, 169	Digallic acid	[26]
3	12.24	183	125, 169	Methylgallate	[25]
4	18.37	635	301, 313, 465, 483	Trigalloyl glucose	[26]
5	34.60	787	301, 617, 635	1,2,3,6-Tetra-*O*-galloylglucose	[27]
6	35.51	335	125, 169, 183	Methyldigallate	[28]
7	36.55	939	301, 425, 465, 617	2,6-bis-*O*-Digalloyl-3-*O*-galloyl –glucose	[5]
8	37.18	1091	939	Hexa-galloyl-glucose	[29]
9	38.69	487	183, 335	Methyltrigallate	[30]
10	38.92	417	151, 179, 285	Kaempferol pentoside	[31]
11	39.17	447	151, 179, 315	Isorhamnetin pentoside	[11]
12	42.73	431	179, 285, 327	Kaempferol rhamnoside	[32]
13	44.63	461	151, 179, 315	Isorhamnetin rhamnoside	[33]
14	47.70	599	315, 447	Isorhamnetin galloyl-pentoside	
15	49.66	585	179, 301, 433	Quercetin galloyl-pentoside	[10]

**Table 2:** In vitro inhibitory activity of *H. campechianum* flower extract against COX1, COX2, LOX and its total antioxidant capacity.

Treatment	COX1	COX2	SI	LOX	TAC
	IC_50_ (µg/mL)			IC_50_ (µg/mL)	U/L
Extract	9.97 ± 0.33	0.59 ± 0.24	16.9	3.70 ± 0.05	33.03 ± 0.66
Celecoxib	15.83 ± 0.66	0.05 ± 0.01	316.6	-	-
Diclofenac	4.23 ± 0.26	0.76 ± 0.07	5.57	2.53 ± 0.23	-
Indomethacin	0.09 ± 0.01	0.76 ± 0.03	0.12	-	-
Zileuton	-	-	-	3.27 ± 0.09	-
Ascorbic acid	-	-	-	-	25.27 ± 1.46

Values are presented as mean ± SD. The COX selectivity index (SI) is calculated as IC_50_ (COX-1)/IC_50_ (COX-2). TAC; total antioxidant capacity, U/L; unit per liter. The experiments were performed in three replicates.

**Table 3:** Effect of *H. campechianum* flowers extract on pyrexia induced by brewer’s yeast in mice.

Experiment	Dose (mg/kg)	Rectal Temperature	Recorded Rectal Temperature after Different Treatments				
			30 min	1 h	2 h	3 h	24 h
Control	-	38.36 ± 0.28	38.60 ± 0.11	38.54 ± 0.10	38.98 ± 0.23	38.84 ± 0.31	38.20 ± 0.16
Extract	200	37.75 ± 0.28	37.33 ± 0.33 *	38.18 ± 0.26	38.70 ± 0.34	38.13 ± 0.21	37.45 ± 0.12
Extract	400	38.28 ± 0.42	37.42 ± 0.47 *	38.92 ± 0.26	38.80 ± 0.13	38.46 ± 0.27	37.34 ± 0.13
Paracetamol	150	38.66 ± 0.18	38.20 ± 0.20	37.36 ±0.36	37.06 ± 0.29 *	36.96 ± 0.24 *	36.50 ± 0.22 *

The values were recorded 18 h after brewer’s yeast injection. Values are presented as mean values ± S.E.M (n = 5), * *p* < 0.01 vs. control values.

**Table 4:** Chronic ulcerogenecity testing of *H. campechianum* flower extract along with two reference drugs, celecoxib and indomethacin.

Experiment	Average Ulcer Number (UN)	Average Severity Score (US)	%Lesion Incidence (UP)	Ulcer Index (UI)
CCI	0.25	0.25	40	2.75
Indomethacin (5 mg/kg)	13.00	1.48	100	24.48
Celecoxib (15 mg/kg)	0.40	0.30	30	3.70
Extract (200 mg/kg)	1.25	0.75	75	9.50
Extract (400 mg/kg)	0.75	0.56	50	6.31

The ulcer index (UI) is the sum of the average ulcer number (UN), average severity score (US), and % lesion incidence (UP) based on the equation (UI = UN + US + UP × 10^−1^).

**Table 5:** Amino acid interactions and scoring functions of the compounds from *H. campechianum* flower extract docked into the p53 DNA binding domain.

Compound	p53 DNA Binding Domain	
	Scoring Function (Kcal/mol)	Amino Acid Interactions
Gallic acid	−11.17	His 178: Coordinate His 178: Ionic Asn 239: H-bond Cys 242: H-bond
Digallic acid	−13.76	His 178: Coordinate His 178: Ionic His 178: H-bond His 179: H-bond Asn 239: H-bond
Trigalloyl glucose	−19.62	His 178: H-bond His 179: H-bond Cys 182: Hydrophobic Ser 183: H-bond Met 243: H-bond
1,2,3,6-Tetra-*o*-galloyl glucose	−12.43	His 178: H-bond His 178: Hydrophobic His 179: H-bond Cys 182: Hydrophobic Met 243: H-bond
Isorhamnetin galloyl-pentoside	−16.53	His 178: H-bond His 178: Hydrophobic His 179: H-bond Asp 184: H-bond
Isorhamnetin rhamnoside	−10.29	His 178: Hydrophobic His 178: H-bond His 179: H-bond Cys 242: H-bond Met 243: H-bond
Isorhmnetin pentoside	−10.03	His 178: Arene-Cation His 179: H-bond Asn 239: H-bond Arg 248: H-bond
Kaempferol rhamnoside	−9.82	His 178: H-bond His 178: Hydrophobic His 179: H-bond Arg 248: H-bond
Kaempferol pentoside	−10.53	His 178: Hydrophobic His 179: H-bond Asn 239: H-bond Cys 242: H-bond
Quercetin galloyl-pentoside	−13.88	His 178: Hydrophobic His 178: H-bond His 179: H-bond Cys 182: Hydrophobic Asn 239: H-bond Met 243: H-bond
Bispicen (positive control)	−9.11	His 178: H-bond His 178: Hydrophobic, Cys 242: Coordinate Met 243: H-bond
