# Correction: Sobeh et al. *Haematoxylon campechianum* Extract Ameliorates Neuropathic Pain via Inhibition of NF-κB/TNF-α/NOX/iNOS Signalling Pathway in a Rat Model of Chronic Constriction Injury. *Biomolecules* 2020, *10*, 386

**DOI:** 10.3390/biom16030347

**Published:** 2026-02-26

**Authors:** Mansour Sobeh, Mona F. Mahmoud, Samar Rezq, Mohamed A. O. Abdelfattah, Islam Mostafa, Amira E. Alsemeh, Assem M. El-Shazly, Aziz Yasri, Michael Wink

**Affiliations:** 1 Institute of Pharmacy and Molecular Biotechnology, Heidelberg University, 69120 Heidelberg, Germany; 2 AgroBioSciences Research Division, Mohammed VI Polytechnic University, Lot 660–Hay MoulayRachid, Ben-Guerir 43150, Morocco; aziz.yasri@um6p.ma; 3 Department of Pharmacology and Toxicology, Faculty of Pharmacy, Zagazig University, Zagazig 44519, Egypt; mona_pharmacology@yahoo.com (M.F.M.); samar_rezq@yahoo.com (S.R.); 4 College of Engineering and Technology, American University of the Middle East, Egaila 54200, Kuwait; mohamed.abdelmoety@aum.edu.kw; 5 Department of Pharmacognosy, Faculty of Pharmacy, Zagazig University, Zagazig 44519, Egypt; islam_mostafa_elbaz@yahoo.com (I.M.); assemels2002@yahoo.co.uk (A.M.E.-S.); 6 Department of Anatomy and Embryology, Faculty of Medicine, Zagazig University, Zagazig 44519, Egypt; dr_amira_2008@yahoo.com

In the original publication [1], there was a mistake in Figures 14 and 15 as published. Figure 14c was previously used in Figure 6c in *Antioxidants* **2019**, *8*, 482 [2]; this occurred because the same control groups were used for experiments that were conducted simultaneously on several plant species. Identical control groups were utilized for both articles in accordance with ethical guidelines aimed at minimizing the number of animals used. Using shared controls allowed us to reduce animal usage while maintaining consistent experimental conditions. Although the control data are the same, the analyses, interpretations, and conclusions presented in each article are entirely distinct. Each manuscript includes its own methodology, statistical analysis, and context to ensure full transparency and scientific rigor. Due to assembly oversight, Figure 15a was mistakenly used again for Figure 15b,e. The corrected Figure 14 and Figure 15 appear below.

The authors state that the scientific conclusions are unaffected. This correction was approved by the Academic Editor. The original publication has also been updated.

## Figures and Tables

**Figure 14:**
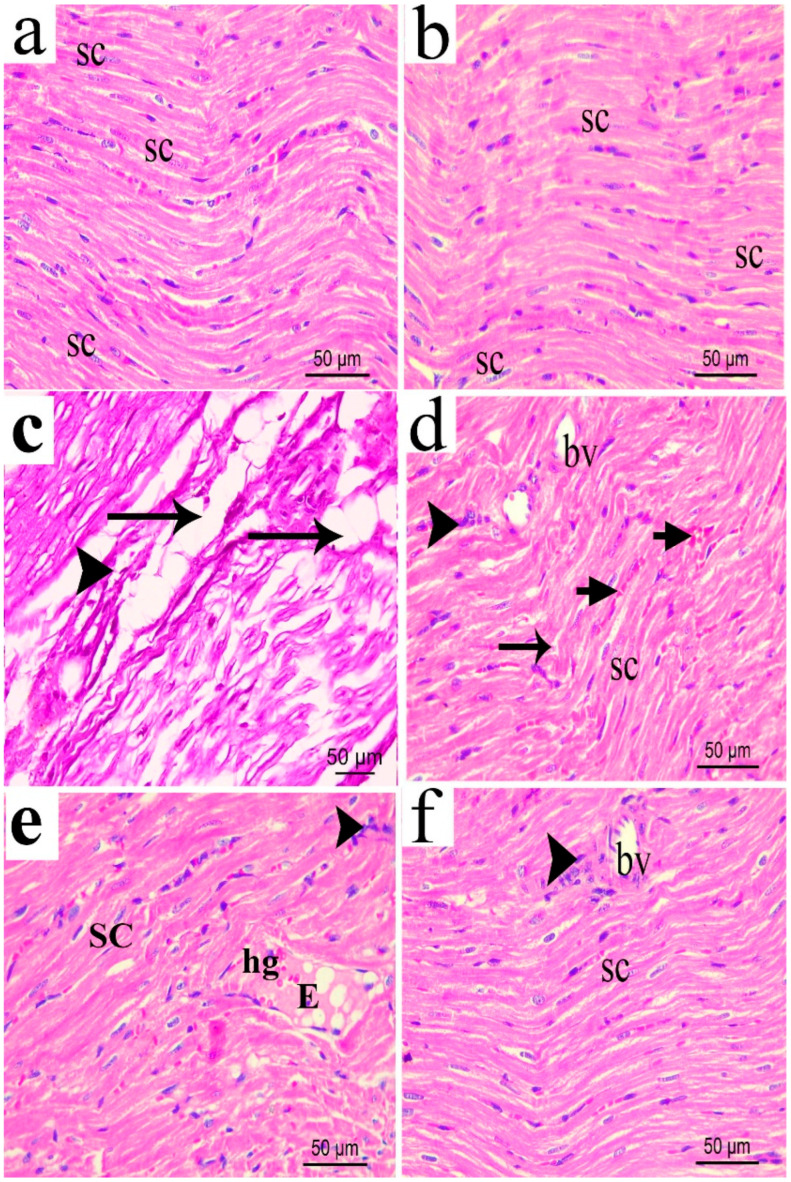
Photomicrograph (H&E × 400) of longitudinal sciatic nerve sections from normal (**a**); sham (**b**); CCI (**c**); pregabalin (**d**); *H. campechianum* flower extract (200 mg/kg); and (**e**) *H. campechianum* flower extract (400 mg/kg) (**f**) rats. Rats were subjected to different treatments for 14 days, after which the animals were sacrificed, and tissues were collected. Arrow and arrowheads illustrate myelin sheet degeneration and mononuclear infiltrating cells, respectively. bv, blood vessel; E, exudate; hg, hemorrhage; SC, Schwan cell nuclei. Scale bar, 50 μm.

**Figure 15:**
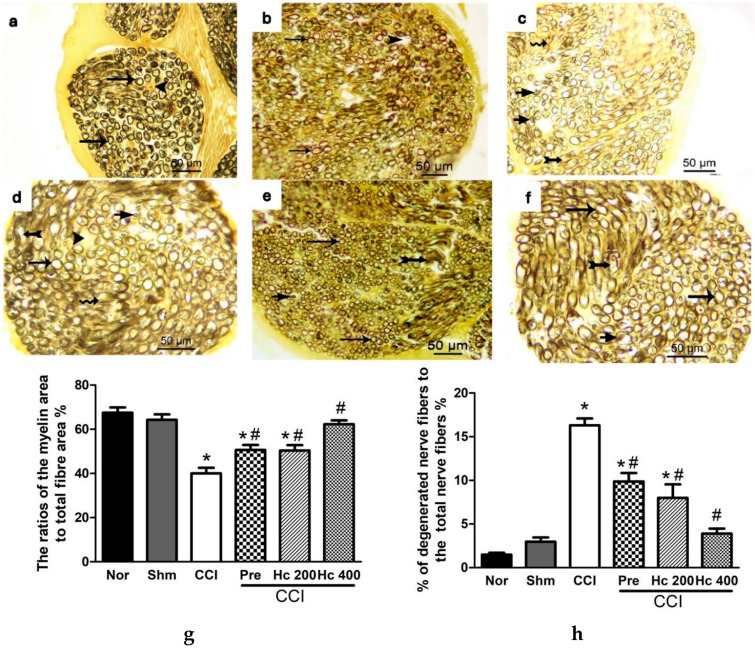
Photomicrographs of sciatic nerve transverse sections stained with osmic acid in (**a**) normal group; (**b**) sham group; (**c**) CCI group; (**d**) pregabalin group; (**e**) *H. campechianum* flower extract (p.o., 200 mg/kg) group; and (**f**) *H. campechianum* flower extract (p.o., 400 mg/kg) group. Arrow, arrowhead, bifid arrow, short arrow, and wavy arrow illustrate the normal myelin sheath concentric lamellar structure, unmyelinated nerve fibres, compromised normal myelin sheath concentric lamellar structure, and invagination of the myelin sheath into the axon and axonal swelling, respectively. Lower panel represents the ratio of myelin sheath to the total nerve area (**g**) and the ratio of degenerated nerve fibres to the total number of nerve fibres (**h**). Data are presented as mean values ± S.E.M. (n = 5–7). * *p* < 0.05, compared to the sham group; ^#^ *p* < 0.05, compared to the CCI group. Scale bar, 50 μm × 400. CCI, chronic constriction injury; Hc, *H. campechianum*; Nor, normal; Pre, pregabalin; Shm, sham.

## References

[1] Sobeh M., Mahmoud M.F., Rezq S., Abdelfattah M.A.O., Mostafa I., Alsemeh A.E., El-Shazly A.M., Yasri A., Wink M. (2020). *Haematoxylon campechianum* Extract Ameliorates Neuropathic Pain via Inhibition of NF-κB/TNF-α/NOX/iNOS Signalling Pathway in a Rat Model of Chronic Constriction Injury. Biomolecules.

[2] Sobeh M., Mahmoud M.F., Rezq S., Alsemeh A.E., Sabry O.M., Mostafa I., Abdelfattah M.A.O., Ait El-Allem K., El-Shazly A.M., Yasri A. (2019). *Salix tetrasperma* Roxb. Extract Alleviates Neuropathic Pain in Rats via Modulation of the NF-κB/TNF-α/NOX/iNOS Pathway. Antioxidants.

